# Comparative genomic and proteomic analyses of two *Mycoplasma agalactiae *strains: clues to the macro- and micro-events that are shaping mycoplasma diversity

**DOI:** 10.1186/1471-2164-11-86

**Published:** 2010-02-02

**Authors:** Laurent X Nouvel, Pascal Sirand-Pugnet, Marc S Marenda, Eveline Sagné, Valérie Barbe, Sophie Mangenot, Chantal Schenowitz, Daniel Jacob, Aurélien Barré, Stéphane Claverol, Alain Blanchard, Christine Citti

**Affiliations:** 1 Université de Toulouse, ENVT, UMR 1225 Interactions Hôtes - Agents Pathogènes, 31076 Toulouse, France; 2 INRA, UMR 1225 Interactions Hôtes - Agents Pathogènes, 31076 Toulouse, France; 3 Université de Bordeaux, UMR 1090 Génomique Diversité Pouvoir Pathogène, 33076 Bordeaux, France; 4 INRA, UMR 1090 Génomique Diversité Pouvoir Pathogène, 33883 Villenave d'Ornon, France; 5 CEA-IG, Genoscope, 91057 Evry Cedex, France; 6 Centre de Bioinformatique de Bordeaux, Université de Bordeaux, 33076 Bordeaux, France; 7 Pôle Protéomique, Centre de Génomique Fonctionnelle Bordeaux, Université de Bordeaux, 33076 Bordeaux, France; 8 Current address: School of Veterinary Science, 250 Princes Highway, Werribee, Victoria 3030, Australia

## Abstract

**Background:**

While the genomic era is accumulating a tremendous amount of data, the question of how genomics can describe a bacterial species remains to be fully addressed. The recent sequencing of the genome of the *Mycoplasma agalactiae *type strain has challenged our general view on mycoplasmas by suggesting that these simple bacteria are able to exchange significant amount of genetic material via horizontal gene transfer. Yet, events that are shaping mycoplasma genomes and that are underlining diversity within this species have to be fully evaluated. For this purpose, we compared two strains that are representative of the genetic spectrum encountered in this species: the type strain PG2 which genome is already available and a field strain, 5632, which was fully sequenced and annotated in this study.

**Results:**

The two genomes differ by ca. 130 kbp with that of 5632 being the largest (1006 kbp). The make up of this additional genetic material mainly corresponds (i) to mobile genetic elements and (ii) to expanded repertoire of gene families that encode putative surface proteins and display features of highly-variable systems. More specifically, three entire copies of a previously described integrative conjugative element are found in 5632 that accounts for ca. 80 kbp. Other mobile genetic elements, found in 5632 but not in PG2, are the more classical insertion sequences which are related to those found in two other ruminant pathogens, *M. bovis *and *M. mycoides *subsp. *mycoides *SC. In 5632, repertoires of gene families encoding surface proteins are larger due to gene duplication. Comparative proteomic analyses of the two strains indicate that the additional coding capacity of 5632 affects the overall architecture of the surface and suggests the occurrence of new phase variable systems based on single nucleotide polymorphisms.

**Conclusion:**

Overall, comparative analyses of two *M. agalactiae *strains revealed a very dynamic genome which structure has been shaped by gene flow among ruminant mycoplasmas and expansion-reduction of gene repertoires encoding surface proteins, the expression of which is driven by localized genetic micro-events.

## Background

Over the last decade, it has become clear that a single bacterial strain is not always representative of the whole species. Moreover, the range of physiological and virulence properties of a given bacterial pathogen most often relies on a particular subset of genes which are responsible for strain-specific lifestyles and may not be equally distributed within the species [1]. Comparative genomics provide a powerful approach to understanding what makes a pathogen but the question of how it can describe a bacterial species is still debated [2]. Within a single bacterial species, mathematical models are predicting the discovery of new genes even after sequencing hundreds of different genomes [3].

The genus *Mycoplasma *includes the smallest self-replicative bacterium, *M. genitalium*, which genome was among the first sequenced [4]. It belongs to the class Mollicutes which regressive evolution from Gram-positive ancestors has been marked by drastic genome downsizing. As a result, contemporary mycoplasmas have limited metabolic capacities and are among the most evolved prokaryotes as they localised on some of the longest branch of the phylogenetic tree of fully sequenced organisms [5]. While our genomic era is accumulating a tremendous amount of data with more than 900 microbial genomes currently available in public databases (Microbial Genome Resource, NCIB), only 15 other mycoplasma genomes have been completed [6-8], including 3 strains of the *M. hyopneumoniae *species [9,10]. This number is surprising low owing the small size of mycoplasma genomes and the several species that are relevant for public and animal health because they are known as pathogenic for man or for a wide range of animals [11].

Recently, genome sequencing of the *M. agalactiae *type strain has shown that a significant portion of its genome (ca. 18%) has undergone horizontal gene transfer (HGT) with members of the phylogenetically distant "mycoides" cluster [12]. This cluster includes a number of mycoplasma species which are, like *M. agalactiae*, important ruminant pathogens and the nature of the exchanged genes suggests that some may play a role in mycoplasma-host interactions. While this first evidence for large HGT in mycoplasmas is offering possible new means for host-adaptation, it has changed our view on the evolution of these minimal bacteria, which is not only driven by gene loss but also by gene flow between organisms sharing a same host [6]. Based on previous studies on *M. agalactiae *genetic diversity, the species appears to be fairly homogeneous with little intra-species genetic variation and most of the isolates resembling the type strain PG2 [13-15]. One of these studies however pointed toward a subset of strains having particular genetic features also found in *M. bovis*, a cattle pathogen closely related to the ovine-caprine *M. agalactiae*, but not in PG2 or in PG2-like strains [14]. One of these particular strains, namely 5632, turned out to harbour (i) a putative **I**ntegrative **C**onjugative **E**lement, ICE, of 27 kpb which occurrence is low in the *M. agalactiae *species but high in *M. bovis *[16], (ii) a different repertoire of genes encoding surface lipoproteins known as the Vpmas [17], and (iii) other genetic elements yet to be characterized [14,18]. While the 5632 and PG2 strains have both been isolated from Spain, data accumulated so far tend to indicate that each stands at one end of the genetic spectrum encountered in the *M. agalactiae *species.

Inter-strain whole genome comparison within a *Mycoplasma *species has been carried once for an important pathogen of swine, *M. hyopneumoniae*. This study has provided evidence of intraspecific rearrangements resulting in strain-specific gene clusters as well as clues to factors related to pathogenicity [10]. To further comprehend the genome plasticity and the mechanisms responsible in mycoplasmas for intra-species genetic diversity, the genome of *M. agalactiae *strain 5632 was fully sequenced and compared in this study with that of the PG2 type strain [12]. Although *M. agalactiae *is an important pathogen of small ruminants [19,20], little is known regarding its virulence or pathogenicity factors. Since all mycoplasmas lack a cell wall, the surface of their membrane acts as the primary interface in the interaction with the host and the environment. For instance, a number of *M. agalactiae *surface components has been shown to stimulate the host humoral response and includes lipoproteins such as the P80 [21], P40 [22], P48 [23], P30 [24] and the Vpma family [25]. Except for P80, all displayed a certain degree of variability in expression either in clonal population as for the phase-variable Vpmas [17,25,26] or among strains as shown for the P30 which promoter is mutated in the P30-negative 5632 strain [24]. In this study, high-throughput identification of proteins expressed under laboratory conditions in *M. agalactiae *strain PG2 and 5632 was performed by a shotgun approach based on 1D SDS-PAGE protein fractionation followed by proteolyses and nanoLC-MS/MS. These proteomic data sets were used to validate genome annotation and, by comparative analyses, to further detect rare events that may be responsible for surface diversification. The combination of comparative genomics with comparative proteomics revealed that both large and localized events are shaping the *M. agalactiae *population structure which one might be much more dynamic than first expected from their reduced genomes.

## Results

### Whole genome and proteome comparison

**Figure 1:**
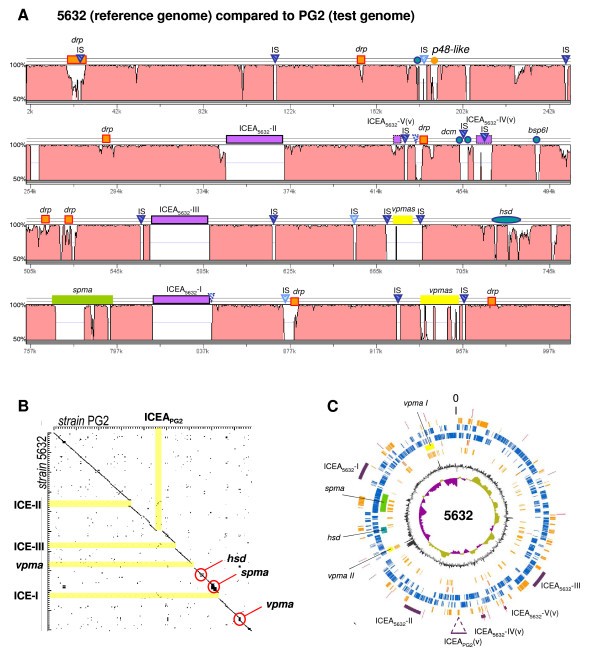
**Overall comparison of *M. agalactiae *genomes from the PG2 and 5632 strains**. **(A) VISTA comparison **[61]. The graph represents the sequence nucleotide identity (in %) using a sliding window of 100 bp and the 5632 genome as a reference. Colored boxes represent gene families or ICE (orange for the *drp *genes, yellow for the *vpma*, green for the *spmas*, and purple for the ICEs); blue triangles insertion sequence (IS) (dark blue for IS*Mag1*, light blue for IS*Mag2*). Filled orange and blue circles represent respectively the p48 lipoprotein gene and CDSs related to restriction-modification systems. Boxes or triangles surrounded with dotted lines indicate pseudogenes or ICE vestiges. **(B) Comparison of CDSs using the MolliGen dot plot alignment **[58]. Each dot represents a blastp hit (threshold 10^-8^) between a CDS of 5632 (ordinates) and a CDS of PG2 (abscises). On axes, the length between two large marks corresponds to 100 kbp. **(C) Circular representation of 5632 genome using the Artemis suite DNAplot **[63]. Outer to inner circles correspond to: circle1, 5632 mobilome with IS in red and ICEs in purple (the position of the unique vestigial ICE of strain PG2 is also indicated); circle 2, CDS predicted as implicated in HGT with mycoplasmas of the "mycoides" group; circle 3, positive strand annotated CDSs; circle 4, negative strand annotated CDSs; circle 5, CDS of interest discussed in the text (color code as in panel 1); circle 6, CDS predicted as lipoproteins; circle 7, percent G+C content (high G+C content in dark grey and low G+C content in light grey); circle 8, GC skew.

Whole genome sequencing of the *M. agalactiae *strain 5632 revealed that it is composed of 1,006.7 kbp and thus, is ca.130 kpb larger than the genome of the PG2 type strain [12] (see Table 1 for general features). The annotated genome of 5632 displays a total of 826 CDS for only 752 in PG2 and whole proteome analyses of both strains identified a global set of 507 as being expressed under laboratory conditions in complex, axenic media (see additional file 1: Table S1). Of these expressed CDS, 184 were detected in only one strain (140 in 5632 and 44 in PG2) and 313 in both. Among these, 139 were annotated as hypothetical, 41 were related to hypothetical ABC transporter while most of the remaining corresponded to house keeping genes. These data indicate that ca. 60% of the *M. agalactiae *predicted CDS products were confirmed by the global proteomic approach. In a recent study by Demina et al. [27], a same proportion of the *M. gallisepticum *annotated proteins was found to be expressed using similar approaches. Whether the remaining annotated CDS of *M. agalactiae *would be expressed or detected under different conditions is not known but it is unlikely that they all correspond to false ORF. Comparison of the two genomes using the MolliGen dot plot, the VISTA and the ACT softwares revealed an almost perfect synteny with no major genome rearrangement but with a number of regions being prominently different (Figure 1). These correspond to (i) mobile genetic elements, (ii) restriction modification systems, and (iii) families of gene encoding surface proteins. As described below, these regions account for most of the difference in CDS content observed in between PG2 and 5632.

**Table 1:** General properties of *M. agalactiae *PG2 and 5632 strains

	PG2	5632
Date of isolation	1952	<1991
Country	Spain	Spain
Source	nk	articulation
Host	caprine	caprine
Genome size (bp)	877,438	1,006,702
G+C (%)	29.70	29.62
Gene density (%)	88.5	88.7
Total number of CDS	752	826
HP (Hypothetical Protein)	138	148
CHP (Conserved HP)	186	150
CDS with predicted function	404	505
Pseudogenes	45	23
rRNAs sets	2	2
tRNAs	34	34
GenBank accession number	CU179680	FP671138
ICE number	(1 vestigial)	3 (+2 vestigial)
Transposase number	1^a^	15
	(+2 pseudogenes)	(+2 pseudogenes)
Genomic DNA digested by:		
*Dpn *I or *Alw *I (sens. to Dam methylation)	Yes	No
*Dpn *II (Dam resistant)	Yes	Yes
Relative colony size^b^	100%	180%

Data were extracted from the MolliGen database http://cbi.labri.fr/outils/molligen/.

^a ^One CDS, MAG3410, was annotated as transposase and was detected by proteomics analysis in this study but no inverted repeat sequences could be found.

^b^Relative colony sizes as defined on agar medium with PG2 as reference. Repeatedly colonies of 5632 were found to be approximately 1.8 times larger than those of PG2.

nk, not known

### Role of the mobilome in *M. agalactiae *genetic diversity and genome plasticity

**Figure 2:**
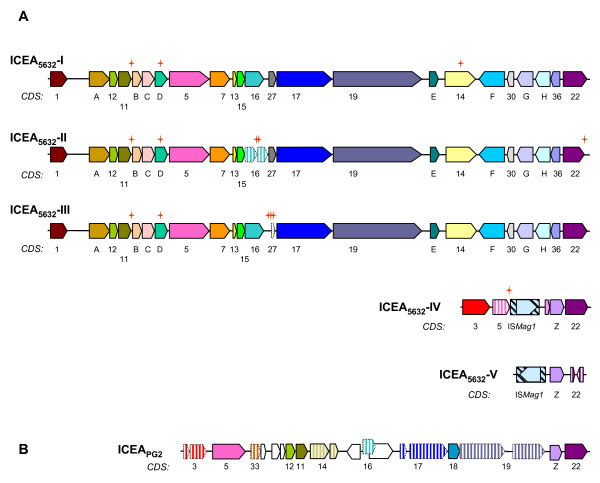
**Comparison of entire and vestigial ICEs found in *M. agalactiae *strains PG2 and 5632**. Schematics represent ICEs encountered in 5632 (A) and in PG2 type strain (B). Large arrows represent CDSs, with homologous CDSs labelled with the same color. CDS nomenclature indicated below arrows is based on the first ICE study in 5632 [16]. ICEA_5632_-I, -II, -III, -IV, -V extend from MAGa7100 to 6880, MAGa2980 to 3220, MAGa4850 to 5060, MAGa4050 to 4010, MAGa3690 to 3670, respectively. ICEA_PG2 _extend from MAG4060 to 3860. Red crosses indicate SNPs or indels in between ICEs from 5632. Insertion sequence elements (IS*Mag1*) are represented by shaded boxes with transposase CDS in light blue. Pseudogenes are represented by hatched colours with dotted lines.

Analyses of the 5632 genome revealed the presence of three large regions (ca. 27 kb) that correspond to an ICE element previously identified in this strain [16]. The three ICE copies were designated ICEA_5632_-I, -II and -III, with ICE-I corresponding to that previously published, and represented a total of about 80 kbp. In addition, two smaller regions designated ICEA_5632_-IV and -V were detected that relate to the degenerated, single ICE form found in strain PG2 [12] and that appear to be ICEs vestiges as suggested by their reduced size and the presence of insertion sequences and pseudogenes. Predicted proteins encoded by these vestiges were designated according to our previous nomenclature (Figure 2). Interestingly, while phylogenetic and BLAST analyses indicate that ICEA_5632_-I to-III are related to the ICEF of *Mycoplasma fermentans *strain PG18 [28], the ICEA_5632_-IV and -V vestiges and the degenerated ICEA_PG2 _are somewhat similar to the ICEC of *M. capricolum*; in particular, they all contain a CDS with no predicted function, CDSZ, that is not found in ICEA_5632_-I to -III nor in ICEF. ICEA_5632_-IV also contains a CDS, CDS3, which is present as a pseudogene in both the ICEA_PG2 _and ICEC but is absent from the large copies ICEA_5632_-I to III. The ICEA copies I to IV also contain two CDS widely conserved in mycoplasma ICEs, CDS22 and CDS5 (the copy -V appear to have a degenerated version of CDS22). ICEA left and right borders of the ICEA have been experimentally defined as GGAA-[ICEA]-TTCC for copy-I [16] and an identical inverted repeat also flanked copies-II and -III. The high level of sequence conservation between the two *M. agalactiae *genomes allowed defining the insertion points of the 3 large ICE copies in the 5632 chromosome which correspond to intergenic regions in PG2. Although ICE insertions do not result in apparent gene disruption, the targeted regions seem to be prone to instability: the copy -I is located next to a conserved insertion sequence (IS) present as a pseudogene in both strains but showing some sequence divergence; the copy -III is also in the vicinity of an IS present only in strain 5632 and which is inserted next to a conserved tRNA gene. The copy -II is inserted next to a predicted lipoprotein gene (MAG2840 or MAGa2970) showing clear sequence divergence as the two predicted proteins have only 66.4% identities. The three ICE copies I-III are flanked by an almost perfect 9 bp direct repeat which is most likely generated during the integration process. Alignment of ICEA-I, -II and -III DNA sequences from the first G of the GGAA repeat to the last C of the TTCC repeat showed that there are highly similar, presenting only 7 to 8 SNPs. This suggests that the copies originated from subsequent excision and integration events, possibly during chromosomal replication or by exchange within the population. SNPs resulted in generating (i) two pseudogenes corresponding to CDS16 of copy II and CDS27 of copy III, (ii) truncation of CDS22 in copy II and (iii) insertion of an Asn codon in a stretch of repeated AAT (poly Asn) in CDS14 of copy I. Overall, 5632 ICEAs account for 21 different CDS that are not present in PG2, with the products of two detected by MS/MS in the Triton-X114 fraction of 5632 (CDS14 and 17).

**Figure 3:**
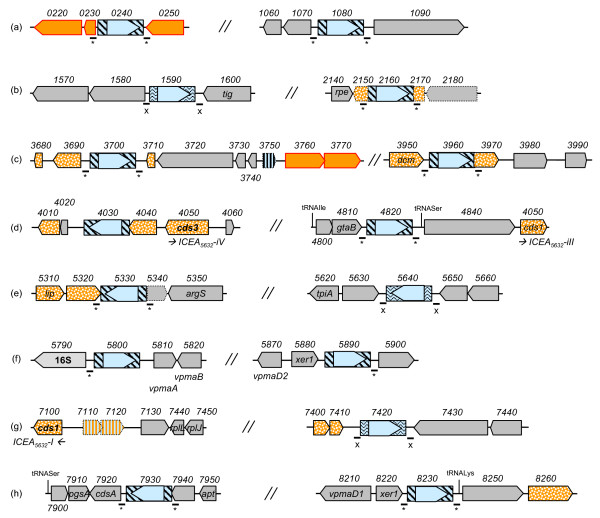
**Location of insertion sequences and their flanking sequences in *M. agalactiae *strain 5632**. Schematics representing genomic regions that flank insertion sequence (IS) elements in strain 5632. Large arrows represent CDSs. IS elements are represented by blue boxes filled with straight lines for IS*Mag1 *or wavy lines for IS*Mag2 *with the transposases being indicated by open arrows filled with light blue. CDSs predicted as implicated in HGT with mycoplasmas of the "mycoides" group are filled by plain orange for *drp *genes or by a dotted orange pattern for the others. MAGa7110 and MAGa7120 that represent a pseudogene of transposase also predicted has implicated in HGT with the "mycoides" group are filled with hatched orange. Short lines with an asterisk (*) or an X below indicates the presence of a 14-nucleotides or of a 25-nucleotides direct repeat flanking IS*Mag1 *or IS*Mag2*, respectively. Pseudogenes are indicated by arrows with dotted lines.

Other mobile elements were found in the 5632 genome but not in PG2 and correspond to multiple copies of two IS elements that both belong to the IS30 family. The location of these elements relative to their flanking CDS is shown in Figure 3. The IS element, IS*Mag1*, has previously been described in some *M. agalactiae *strains [29] and an isoform was also described in *M. bovis *(named IS*Mbov1*) [30]. In 5632, this element occurs in 12 copies with 10 that localized either next to genomic islands encoding a repertoire of variable surface lipoproteins (see MAGa5890, MAGa5800 and MAGa8230) or to regions associated with HGT (see circles 1 and 2 in Figure 1C). The second type of IS, IS*Mag2*, resembles IS*Mbov6 *recently described [31] and is found only in three copies, none of which seems to truncate or disrupt a CDS.

*In silico *analyses further indicate that IS*Mag1 *occurrence most likely disrupts gene expression in only three cases. In the first case, the insertion has taken place in between a *dcm *gene encoding a Cytosine-specific DNA-methyltransferase Sau96I (MAGa3950) and a Type II specific deoxyribonuclease *sau96I-like *gene (MAGa3970). Both genes are absent from PG2 but are found next to each other in *M. mycoides *subsp. *mycoides *SC in which they most likely occur as an operon. These genes are highly similar to those found in 5632 with ca. 85% and 78% similarity, respectively, with higher divergence in the N-terminal of 5632 *sau96I-like *gene. Interestingly, a global proteomic approach (see below and additional file 1: Table S1) detected several specific peptides of the cytosine-specific methyl transferase encoded by MAGa3950 but none corresponding to the *sau96I-like *gene supporting the hypothesis that the IS occurrence at its 5'end may affect its expression. In the second case, IS insertion at the 3' end of MAGa4040 would result in truncating the product by more than 50% when compared to the situation found in PG2. The third case relates to MAGa5320 and MAGa5350 which are separated by an IS and which have been annotated as two distinct pseudogenes because they are highly similar to either the N- or the C-terminal part of the *Mycoplasma capricolum *subsp. *capricolum *glycosyl transferase (MCAP0063). In PG2, homologs to MAGa5320 and MAGa5350 also exist as pseudogenes although no IS is involved.

Finally, two vestiges of transposase having similarities with that of IS*Mmy1 *of *M. mycoides *subsp. *mycoides *SC were detected in the 5632 genome; one located next to an ICE element while the other was found next to a truncated hypothetical protein that displays a DUF285 motif (see below) and that is predicted to have undergone HGT with member of the "mycoides" cluster species.

**Figure 4:**
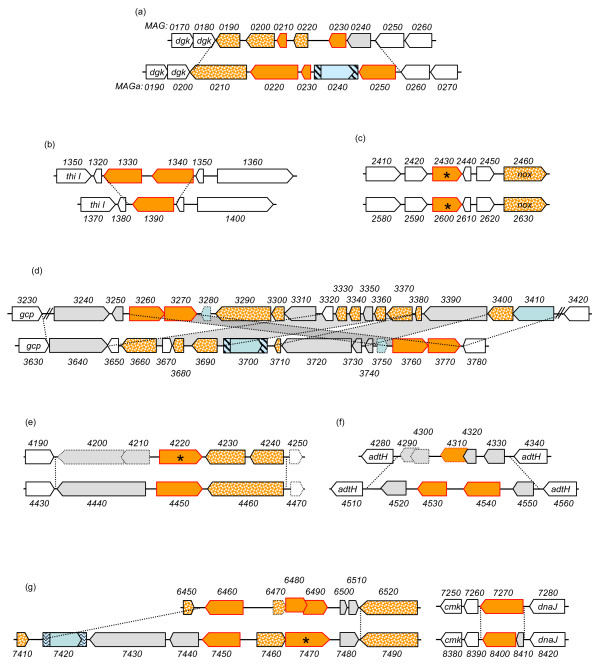
**Comparison of *M. agalactiae *PG2 and 5632 revealed that the *drp *loci are sequence reservoirs for strain genetic and surface diversity**. Schematics representing the comparison of genomic regions containing *drp *genes in strains PG2 (MAG, upper schematics) and 5632 (MAGa; lower schematics). Large arrows represent (i) CDSs corresponding to *drp *genes (filled by plain orange with red outlines) (ii) CDSs others than *drp *and predicted as implicated in HGT with mycoplasmas of the "mycoides" group (filled by dotted orange) or (iii) CDSs conserved between PG2 and 5632 (filled by plain white). Insertion sequence elements are represented as in Figure 3. Drps detected by LC-MS/MS are labelled by an asterisk (corresponding in PG2 and 5632, respectively, to MAG2430 and MAG4220, MAGa2600 and MAGa7470). Pseudogenes are represented by large arrows with dotted lines. Limits of variable regions are indicated by dotted lines connecting the orthologous regions in both strains. Numbers above and below CDS correspond to MAG or MAGa mnemonics.

In most cases, IS elements were flanked by direct repeated sequences of 14 nt for IS*Mag1 *and of 25 nt for IS*Mag2 *that indicated a single IS insertion event. Exceptions were found for IS elements (MAGa5800 and MAGa5890) located next to the *vpma *gene family as previously described [17] and was also observed here for the IS insertion located at the 3' end of MAGa4040 suggesting that further genomic rearrangements have occurred in this area. Indeed, this region has been described above as a putative vestige of ICE integration. Finally, a single transposase gene which product was detected by proteomic analyses (MAG3410; see also Figure 4) is found in PG2 but not in 5632. This transposase has some similarities (46.7%) with an IS*Mmy1 *transposase of *M. mycoides *subsp. *mycoides *SC, but no flanking repeated sequence could be readily identified.

These data indicate that ca. 76% of the additional genomic material of 5632 is composed of mobile genetic elements when compared to PG2. This represents 10% of the genome, yet these do not lead to major genome rearrangement. Overall, 5632 has 95 additional CDS, 72 of which correspond to CDS of ICE or transposase. Among the remaining, 8 relate to the *spma *family (see below), 11 to hypothetical products (including 4 detected by LC-MS/MS) and 4 to restriction modification systems (RM) (Table 2).

**Table 2:** Restriction/Modification products comparison between strains PG2 and 5632

MAGa^a^	MAG^b^	Product	Similarity (%)	MS/MS^c ^5632	MS/MS^c ^PG2	Comments
MAGa1570	MAG1530	Type III R/M system:Methylase	75.3	+	+	
MAGa1580			77.6	+		

MAGa1770	MAG1790	DNA methylase	97.8	-	-	

MAGa2070	MAG2070	DNA methylase	98.9	+	-	

MAGa2700	*MAG2550*	Adenine-specific DNA methyltransferase	65.8	-^d^	-	Pseudogene in PG2
	*MAG2560*			-		

MAGa2710	*MAG2570*	Type II restriction endonuclease **	46.1	-	-	Pseudogene in PG2
	*MAG2580*			-		

No homolog	MAG3310	CpG DNA methylase	na	na	-	

No homolog	MAG4030	Conserved hypothetical protein	na	na	-	BBH:*Mmm *SC - Putative C5 methylase (40%)

MAGa4470	MAG4250	Pseudogene of CpG DNA methylase (N-terminal)	83.4	-	-	

MAGa4480	MAG4260	Pseudogene of CpG DNA methylase (C-terminal)	94.7	-	-	

MAGa6280	MAG5640	Type I R/M system specificity subunit	75.0	-	+^d^	Locus *hsd*

MAGa6290	MAG5650	Modification (Methylase) protein of type I restriction-modification system HsdM	98.3	+	-	Locus *hsd*

MAGa6310	MAG5680	Type I R/M system specificity subunit	32.4	-	+^d^	Locus *hsd*

MAGa6330	*MAG5700*	HsdR, R/M enzyme subunitR	95.0	+	-	Pseudogenes in PG2
	*MAG5710*			-		

MAGa6340	MAG5720	Type I R/M system specificity subunit	30.9	+	-	Locus *hsd*

MAGa6350	MAG5730	Modification (Methylase) protein of type I restriction-modification system HsdM	90.3	+	+	Locus *hsd*

MAGa7650	MAG6680	Modification methylase	97.6	+	-	Modification methylase

MAGa3200 MAGa5050 MAGa6900	No homolog	CDSH	na	-	na	BBH: 92.0% with MCAP0297 - *Mcap *- adenine-specific DNA methylase

MAGa4250	No homolog	Modification methylase Bsp6I	na	+	na	BBH: 81.7% *Bacillus sp. bsp6 *IM Modification methylase Bsp6I

MAGa4260	No homolog	Type II restriction enzyme Bsp6I	na	+	na	BBH:55.1% *Bacillus sp bsp6 *IR Type II restriction enzyme Bsp6I

MAGa3950	No homolog	Cytosine-specific methyltransferase	na	+	na	BBH: *Mmm *SC MSC_0216 *dcm *Cytosine-specific DNA-methyltransferase Sau96I

MAGa3970	No homolog	Type II site-specific deoxyribonuclease, *sau*96I-like	na	-	na	BBH: *Mmm *SC MSC_0215 *sau*96I Type II site-specific deoxyribonuclease

^a^, CDS of *M. agalactiae *strain 5632 (MolliGen Mnemonic).

^b^, CDS of PG2 (MolliGen Mnemonic), pseudogenes are indicated in italic.

^c^, Proteomic analyses (see materials and methods): (+) indicates that peptides were detected by MS/MS for the corresponding CDS, suggesting expression of the corresponding gene, (-) indicates that no specific peptides were detected for the corresponding CDS.

^d^, only one peptide detected.

^e^, MAG5640 and MAG5680 have common peptides.

BBH, Best Blast Hit; *Mmm *SC, Mycoplasma *mycoides *subsp. *Mycoides *SC; *Mcap*, *M. capricolum *subsp. c*apricolum*; na, not applicable; R/M, Restriction/Modification

### Protection from DNA degradation and invasion

While genetic transformation of strain PG2 [26,32] has become a standard protocol in our laboratory, attempts to transform 5632 in the same or modified conditions repeatedly failed. As well, 5632 chromosomal DNA is resistant to two type II restriction enzymes, *Alw *1 (GGATCNNNN↓N) and *Dpn *II (↓GATC), that are sensitive to Dam methylation and restrict DNA extracted from PG2. Conversely, 5632 DNA is digested by *Dpn *I (GA↓TC) which cleaves only when the adenine of its recognition site is methylated (data not shown). This suggests that the two strains contain a different set of restriction-modification (RM) systems and, indeed, 5632 contains four additional CDS that encode two type II RM systems, each composed of a putative restriction enzyme and its corresponding methylase. The first one is similar to the *Bacillus sp. *Bsp61I RM system while the other resembles that of the Sau96I-like found in *M. mycoides *subps. *mycoides *SC as mentioned above. Indeed, phylogenetic tree reconstructions, although not fully demonstrative, suggested that the Bsp61I RM system has most likely been acquired by HGT from *Firmicutes *other than *Mollicutes *while the Sau96I-like system has probably been exchanged with members of the "mycoides" cluster. Further detailed comparative analyses revealed that 5632 is better equipped than PG2 in terms of RM systems and more specifically in DNA methylases. As indicated in Table 2, 5632 encodes for 11 putative DNA methylases of which 9 are expressed under laboratory conditions while for PG2 this number is only 8 with the expression of 3 being detected. Interestingly, one methylase gene seems to have been duplicated in 5632 (MAGa1570 and MAGa1580) when compared to PG2. The two paralogs differ from each other and from their PG2 ortholog mostly in their central part (ca. aa205-aa400) which is known to contain the N6_N4_Mtase domain (PF01555 in Pfam). Whether this provides the corresponding enzymes with different specificity is not known. Both strains display a locus of six genes (MAGa6280 to MAGa6350, MAG5640 to MAG5730) with homology to type I RM systems that were designated *hsd *and are composed of (i) two *hsdM *genes coding for two almost identical modification enzymes which would methylate specific adenine residues, (ii) three *hsdS *genes each coding for a distinct RM specificity subunit (HsdS) that shares homologies with each other (between 50 to 97% similarities) and (iii) one *hsdR *gene encoding a site-specific endonuclease (HsdR). Interestingly, in PG2, the *hsdR *gene is disrupted by the insertion of two nucleotides in a polyA tract localised in the middle of the gene that results in a premature stop codon. This is in agreement with the detection in 5632 but not in PG2 of peptides specific of the HsdR enzyme. In mycoplasma, such polyA tracts have often been involved in high-frequency variation in expression [33]. Finally, the *hsd *locus also contains a hypothetical CDS whose product is highly similar to a phage family integrase of *Bifidobacterium longum *[34] and motifs found in molecules involved in DNA recombination and integration. In *M. pulmonis*, the *hsd *locus has been shown to undergo frequent DNA rearrangements but the gene encoding the putative recombinase is located elsewhere in the genome [35,36]. If the *hsd *locus of *M. agalactiae *is functional then it is worth noting that its *hsdS *sequences diverge between the two strains suggesting that recombinase-mediated DNA rearrangements could modulate the specificity of the system. Attempts to demonstrate DNA rearrangements of the *hsd *using basic molecular approaches failed. Whether this is due to the difficulties in finding specific sequence signatures that would demonstrate recombination is not known.

### The flexible gene pool: towards a highly dynamic surface architecture

Comparison of the two *M. agalactiae *genomes further revealed that strains 5632 and PG2 contain 103 and 67 CDS predicted to encode lipoproteins, respectively. Proteomic analyses further confirmed the expression of more than 50% of these CDS for both strains, with at least 56 being expressed in 5632 and 43 in PG2, all but one (MAGa5190) being detected in Triton X-114 (see Table 3 for a detailed list). In most cases, these differences are linked to genes present in one strain but not in the other (i.e. genes belonging to 5632-ICE and encoding lipoproteins such as CDS14) and to a previously well-characterised gene family, the *vpma*. This family encodes related, phase-variable, lipoproteins [25] and account for 23 CDS in 5632 but only 6 in PG2. As previously reported, all *vpma *genes except two (*vpmaK *and *vpmaL*) were shown to be expressed at one point during *in vitro *propagation of 5632 [17].

**Table 3:** Lipoproteins and MS/MS detection in Tx-114 phase

MAGa^a^	MAG^b^	Gene name	Product	Tx 5632^c^	Tx PG2^c^	Comments
MAGa0140	MAG0120		Conserved hypothetical protein, predicted lipoprotein, P48	+	+	
MAGa0380	MAG0380	*oppA*	Oligopeptide ABC transporter, substrate-binding protein (OppA), predicted lipoprotein	+	+	
MAGa1090	MAG1000		Conserved hypothetical protein, predicted lipoprotein	+	+	
MAGa1140	MAG1050		Hypothetical protein, predicted lipoprotein	+	+	
MAGa1490	MAG1450		Conserved hypothetical protein, predicted lipoprotein	+	+	
MAGa1550	MAG1510		Hypothetical protein, predicted lipoprotein	+	+	
MAGa1620	None		Conserved hypothetical protein, P48-like	**+**	na	No signal peptide and lipobox except if variation in the length of a poly G_10 _(+/-1) upstream the chosen start
MAGa1680	MAG1670		Conserved hypothetical protein, predicted lipoprotein	+	+	
MAGa1980	MAG1980		Hypothetical protein, predicted lipoprotein	+	+	
MAGa1980	MAG1980		Hypothetical protein, predicted lipoprotein	+	+	
MAGa2000	MAG2000		Hypothetical protein, predicted lipoprotein	+	+	
MAGa2330	MAG2220		Conserved hypothetical protein, predicted lipoprotein	+	+	
MAGa2500	MAG2340		Conserved hypothetical protein, predicted lipoprotein	+	-	Not predicted as lipoprotein in PG2 due to variation of the length of a poly A (A_6 _in PG2, A_7 _in 5632)
MAGa2510	MAG2350		Hypothetical protein, predicted lipoprotein	+	+	
MAGa2570	MAG2400		Hypothetical protein, predicted lipoprotein	+	+	
MAGa2580	MAG2410		P40, predicted lipoprotein	+	+	
MAGa2600	MAG2430		Conserved hypothetical protein, predicted lipoprotein, DUF285 family	+	+	
MAGa2670	MAG2510		Hypothetical protein, predicted lipoprotein	+	+	
MAGa2690	***MAG2540 +MAG2530***		Hypothetical protein, Vpma-like, predicted lipoprotein	+	+	For PG2, only MAG2540 was detected and corresponds to the 5'coding end of a pseudogene in PG2
MAGa2740	MAG2610		Hypothetical protein, predicted lipoprotein	+	+	
MAGa2820	MAG2690	*phnD*	Alkylphosphonate ABC transporter, substrate-binding protein, predicted lipoprotein	+	+	
MAGa2970	MAG2840		Conserved hypothetical protein, predicted lipoprotein	+	+	
MAGa3160	None		CDS14	+	na	ICE
MAGa3250	MAG2870		Conserved hypothetical protein, predicted lipoprotein	+	-	None
***MAGa3330 +MAG3340***	MAG2950		Hypothetical protein, predicted lipoprotein	-	+	Variation of the length of a poly C (C_9 _in PG2, C_8 _in 5632) downstream of MAGa3330 may be responsible for frameshifting
MAGa3640	MAG3240		Conserved hypothetical protein, predicted lipoprotein	+	+	Not predicted as lipoprotein in PG2
MAGa3820	***MAG3460***		Hypothetical protein, predicted lipoprotein	+	-	Variation of the length of a poly G (G_8 _in PG2, G_9 _in 5632) upstream of MAG3460 may be responsible for frameshifting
MAGa3830	MAG3470	*p30*	P30, predicted lipoprotein	-	+	Mutation in the *p30 *promoter region of 5632 (Fleury *et al.*[24])
MAGa3980	MAG3590		Hypothetical protein, predicted lipoprotein	-	+	None
MAGa3990	MAG3600		Hypothetical protein, predicted lipoprotein	+	+	
MAGa4680	MAG4460		Conserved hypothetical protein, predicted lipoprotein	+	+	
MAGa5010	None		CDS14	+	na	ICE
MAGa5110	MAG4640		Conserved hypothetical protein, predicted lipoprotein	-	+	None
MAGa5190	MAG4720		Conserved hypothetical protein, predicted lipoprotein	-	-	MAGa5190 was detected in the insoluble pellet
MAGa5210	MAG4740		Hypothetical protein, predicted lipoprotein	+	+	
MAGa5420	***MAG4960 +MAG4950***		Conserved hypothetical protein, predicted lipoprotein	+	-	MAG4960+MAG4950 previously annotated as pseudogenes and detected in total proteins but not in detergent TX-114 phase
MAGa5490	None^d^		Hypothetical protein, predicted lipoprotein	+	+	CDS missed during annotation of PG2 (nt 586236 to 585832)
MAGa5500	MAG5030		P80, predicted lipoprotein	+	+	
MAGa5510	MAG5040		Conserved hypothetical protein, predicted lipoprotein	+	+	
MAGa5560	MAG5080		Hypothetical protein, predicted lipoprotein	+	+	
MAGa5630	MAG5150		Hypothetical protein, predicted lipoprotein	+	+	Not predicted as lipoprotein in PG2 due to the start chosen during annotation
MAGa5830	None	*vpmaC*	Variable surface lipoprotein C (VpmaC)	+	na	Duplicated (MAG8080)
MAGa5850	None	*vpmaE*	Variable surface lipoprotein E (VpmaE)	+	na	Duplicated (MAGa8090)
MAGa5860	None	*vpmaF1*	Variable surface lipoprotein F1 (VpmaF1)	+	na	Duplicated (MAGa8170)
MAGa5870	None	*vpmaD2*	Variable surface lipoprotein D2 (VpmaD2)	+	na	Duplicated (MAGa8120)
MAGa6560	MAG5910		5'Nucleotidase, predicted lipoprotein	+	+	
MAGa6940	None		CDS14	+	na	ICE
MAGa7130	MAG6170		Hypothetical protein, predicted lipoprotein	+	+	
MAGa7160	MAG6200		Hypothetical protein, predicted lipoprotein	+	+	
MAGa7470	***MAG6490 +MAG6480***		Hypothetical protein, predicted lipoprotein, DUF285 family	+	-	Variation of the length of a poly A (A_6 _in 5632, A_7 _in PG2) may be responsible for frameshift
MAGa7490	MAG6520		Conserved hypothetical protein, predicted lipoprotein	+	+	
MAGa8040	None	*vpmaG*	Variable surface lipoprotein G (VpmaG)	+	na	*vpma *family
MAGa8050	None	*vpmaF2*	Variable surface lipoprotein F2 (VpmaF2)	+	na	*vpma *family
MAGa8060	MAG7070	vpmaX*	Variable surface lipoprotein X (VpmaX)	+	+	*vpma *family
MAGa8070	MAG7060	vpmaW*	Variable surface lipoprotein W (VpmaW)	+	+	*vpma *family
MAGa8100	None	*vpmaB*	Variable surface lipoprotein B (VpmaB)	+	na	Duplicated (MAGa8100)
MAGa8110	None	*vpmaA*	Variable surface lipoprotein A (VpmaA)	+	na	Duplicated (MAGa8110)
MAGa8150	None	*vpmaH*	Variable surface lipoprotein H (VpmaH)	+	na	*vpma *family
MAGa8160	None	*vpmaI*	Variable surface lipoprotein I (VpmaI)	+	na	*vpma *family
MAGa8180	None	*vpmaJ*	Variable surface lipoprotein J (VpmaJ)	+	na	*vpma *family
MAGa8210	None	*vpmaD1*	Variable surface lipoprotein D1 (VpmaD1)	+	na	Duplicated (MAGa5840)
MAGa8260	MAG7130		Hypothetical protein, predicted lipoprotein	+	-	Not predicted as lipoprotein in PG2 due to a point mutation: TAA (ochre) ↔TCA (serine))
None	MAG1570		Hypothetical protein	-	+	No signal peptide and lipobox except if variation of the length of a poly G_9 _(+/-1) next to the chosen start
None	MAG7050	*vpmaV*	Variable surface lipoprotein V (VpmaV)	na	+	*vpma *family
None	MAG7080	*vpmaY*	Variable surface lipoprotein Y (VpmaY)	na	+	*vpma *family
None	MAG7090	*vpmaU*	Variable surface lipoprotein U (VpmaU)	na	+	*vpma *family
None	MAG7100	*vpmaZ*	Variable surface lipoprotein Z (VpmaZ)	na	+	*vpma *family

^a ^CDS of *M. agalactiae *strain 5632 (Molligen Mnemonic).

^b ^CDS of PG2 (Molligen Mnemonic), pseudogenes are indicated in italic and bold.

^c ^Peptides detected by MS/MS in the Triton-X114 phase (Tx) (see the Methods section): (+) indicates that peptides corresponding to CDS were detected, suggesting expression of the corresponding gene, (-) indicates that no peptides corresponding to CDS were detected.

^d ^CDS detected in proteomic but for which no Mnemonic was defined because it was missed during the annotation of the PG2 genome [12].

na, not applicable.

Hypothetical related surface lipoproteins are encoded by two other gene families: the so-called *drp *(for DUF285 related proteins) and the *spma *(surface protein of *M. agalactiae*). Unlike the *vpma*, CDS encoding products with DUF285 motifs are scattered on the chromosome, with both strains having a similar size-repertoire composed of 12 CDS identified as Drp (and one pseudogene) in PG2 and 13 in 5632. One particularity of this family is that it belongs to the gene pool that underwent HGT with members of the "mycoides" cluster. Comparison of 5632 with PG2 revealed that they often localized in regions that vary the most between the two strains (Figure 4). Except for one (MAGa2580 to MAGa2630), all 5632 *drp *loci present a different organization when compared to PG2 that reflects the occurrence of complex DNA rearrangements (i.e. locus MAGa3630 to MAGa3780), of additional IS elements or CDS in 5632 (i.e. locus MAGa7410 to MAGa7490), and/or of pseudogenes in PG2 (i.e. MAG4200 and 4210). Interestingly, only two Drp proteins were detected by proteomic LC-MS/MS. One was expressed in both strains (MAG2430 and MAGa2600) and is encoded at the same locus (Figure 4) while the other corresponded to MAG4220 in PG2 or to MAGa7470 in 5632 that are located at two different loci. Interestingly, the homolog MAGa7470 occurs as a pseudogene in PG2 because of a difference in the length of polyA tract that creates by frameshifting a premature stop codon. Concerning MAG4220 and its counterpart in 5632, MAGa4450, there is no apparent molecular feature that could account for their difference in expression. These two products differ slightly from the rest of the family in that they both lack the sequence needed for prolipoprotein recognition and lipid modification, known as the lipobox and usually located at the C-terminal of their signal peptide.

**Figure 5:**
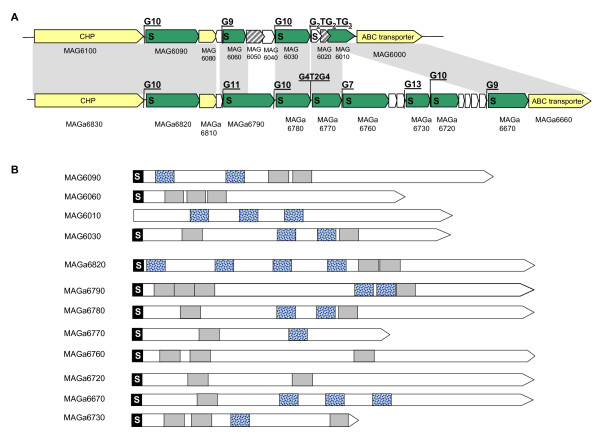
**The *M. agalactiae *5632 strain contains an extended *spma *repertoire**. Schematics representing the genomic organization of the *spma *loci in strains PG2 and 5632 (A) and the structural features of the corresponding *spma *gene products in both strains (B). In panel A, CDS corresponding to *spma *genes are filled in green. The S letter represents sequence corresponding to a signal peptide. Other CDSs conserved between PG2 and 5632 are filled by light yellow. Tracks of repeated nucleotides (Gn, where n is the number of residues) found before *spma *coding sequences are also indicated above the line. In panel B, predicted Spma proteins are represented schematically by large arrows beginning generally with a homologous amino-acid leader sequence (black boxes labelled S) followed by regions that have homology between *spma *gene products or that are repeated within the same product (blue dotted and grey boxes).

Comparison of the 5632 and PG2 genomes revealed one particular locus composed of several putative CDS encoding (i) a similar N-terminal signal peptide followed by a highly conserved lipobox and (ii) particular amino-acid motifs that are repeated within a particular product. This gene family was further designated as *spma *for "surface protein of *M. agalactiae*" and is larger in 5632 with 8 *spma *genes and only 4 in PG2. Analyses of the two *spma *loci indicate that *spma *genes present in 5632 but not in PG2 have orthologs in the "mycoides" cluster only. More specifically, *M. mycoides *subsp. *mycoides *LC strain GM12 [37,38] and *M. capricolum *subsp. *capricolum *contain 5 and 1 genes, respectively, that encode putative lipoproteins resembling 5632-Spmas and carrying the motif 3. Although this question cannot be formally addressed by phylogenetic tree reconstruction, the *spma *sequence comparison suggests that these genes are part of the gene pool which has been exchanged between *M. agalactiae *and members of the "mycoides" cluster. The proteomic approach taken in this study failed to detect any of the Spma products in one or the other *M. agalactiae *strains. Whether these proteins have been missed by this approach or whether they are not expressed or expressed under different conditions remains to be assessed. A stretch of polyG was found at the 5' untranslated region of each putative *spma *gene (Figure 5). This last feature is unusual in mycoplasmas that have a low G+C content and is particularly striking in the 5632 *spma *locus which displays 8 polyG tracts with one containing up to 13 G residues. Whether these control or affect the transcription of downstream genes is not known but homopolymeric tracts of residues have often been associated with products whose expression is phase variable in mycoplasmas.

**Figure 6:**
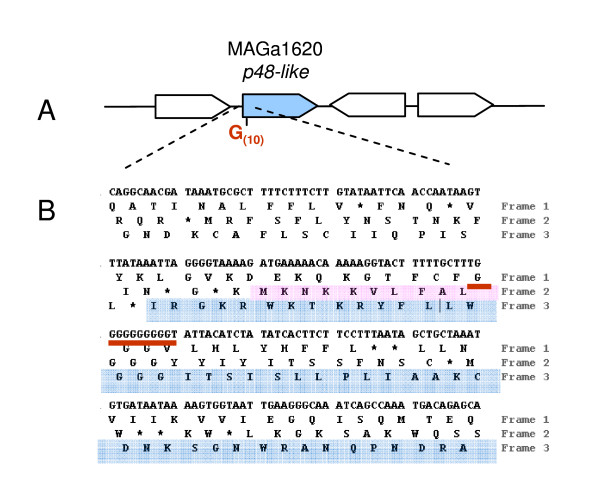
**Analysis of the p48-like sequence of *M. agalactiae *5632 suggests a mechanism for phase variation**. Schematic represents the *p48-like *genomic region (A). CDSs are represented by large arrows with MAGa1620 corresponding to *p48-like *gene filled in blue. Translation of the DNA region flanking the polyG track is given in the three frames (B). The polyG tract suspected to vary in length (G10 +/-1) is underlined by a bold red bar. The putative beginning of a P48-like lipoprotein with an entire signal peptide sequence is shaded in red while the current annotated MAGa1620 open reading frame is in blue. Global amino-acid alignment results obtained with Needle (program available at http://www.ebi.ac.uk/Tools/emboss/align/) between the P48-like of *M. agalactiae *5632 and the P68 lipoprotein of *M. bovis *PG45 for which a similar polyG tract was previously described [48], are of 89.3% (identity) and 92.1% (similarity).

Interestingly, polyG tracts were found elsewhere in the genome of 5632, again at the 5' end of gene encoding putative surface protein. For instance, the conserved hypothetical P48-like product encoded by MAGa1620 displays a high similarity with the P48 lipoprotein and is detected by MS/MS in the triton detergent phase although its gene does not contain a proper signal peptide followed by a lipobox. Careful examination of the 5' end of the P48-like coding sequence revealed a stretch of 10 Gs and a ribosomal frameshift at this position where the deletion of one G would generate an in-frame signal peptide followed by a lipobox (Figure 6). These data suggested that a mechanism based on polyG (or C) being prone to ribosomal shifting or to mutation could also account in several cases for the difference between the two strains in lipoproteins detected in Triton-X114 by proteomic analyses (see Table 3).

## Discussion

Whole genome sequencing of *M. agalactiae *strain 5632 revealed that it contains an additional 95 genes representing an extra-130 kbps when compared to the PG2 type strain. For organisms that have a small genome size such as mycoplasmas, this is a rather significant feature. The additional material is mostly composed of repeated elements, so that our knowledge of the *M. agalactiae *pan-genome has been enriched by 39 new genes. A large portion of those, more specifically 23, is present in ICEs or corresponds to IS. Recent mathematical models by Tettelin *et al. *[39] show that the pan-genome of the mollicute *Ureaplasma urealyticum *is limited, based on the draft sequences of nine strains. This implies that the sequencing of additional strains might not significantly increase our knowledge of this species unless it is targeting a specific biological question [40]. Although *U. urealyticum *is a human pathogen and has a genome slightly smaller (ca. 750 kbp), the same observation may apply to the *M. agalactiae *species as indicated by the low number of new genes discovered in our study. Thus, sequencing additional *M. agalactiae *strains might bring little more information on the global coding capacity of this organism.

Overall, data obtained here and elsewhere indicate that about 10% of the 5632 genome is highly dynamic in that large regions corresponding to ICE can excise [16] and, theoretically, relocate elsewhere or be transferred to a recipient cell during conjugation, if such event is further shown to occur in this species. The two ICE's vestiges, ICE IV and V, represent scars of past ICE insertions followed by a progressive decay. Interestingly, these more resemble the larger ICE vestige of PG2 or the ICE of *M. capricolum *subsp. *capricolum *than the three entire ICE copies of 5632 suggesting that this strain may have, at one point, hosted two types of ICEs. These data indicate that the circulation of ICEs in some strains might not be such a rare event. The presence of ICE circular forms in 5632 [16] together with the low number of SNPs detected between the three copies indicate that multiple ICE insertions are recent. The mechanisms underlying ICE insertion, excision and putative transfer in mycoplasmas have yet to be investigated, but recent studies on ICE elements in Gram-positive bacteria suggest that these events can be under the control of sophisticated regulation systems in response to changing environmental conditions such as stress or population density [41]. The finding of ICE in *M. agalactiae *and members of the "mycoides" cluster together with evidence of HGT in between these species further raised the prospect that these simple bacteria could conjugate. So far, a single report has supported the occurrence of conjugation in mycoplasmas by showing the exchange of genetic material in between *M. pulmonis *cells via a mechanism resistant to DNAse [42]. The idea that this phenomenon might be more common among mycoplasmas than first expected is very exciting because, if occurring, it would change the way we see the evolution of these so called "minimal organisms".

Although smaller in size than ICE, IS elements as a whole represent a dynamic potential for the genome because of their copy number. In other bacteria, their contribution to genome plasticity and dynamics is well known [43]. Here, no major DNA inversion or rearrangement was detected between the two *M. agalactiae *genomes that could be associated to IS except for two cases. As previously shown by our group, the first one refers to the duplication in 5632 of the single *vpma *cluster of PG2 that has been most likely driven by IS elements and that resulted in 5632 having extended possibilities for surface diversification when compared to PG2 [17]. The second case refers to a region which organization significantly differs in between PG2 and 5632 (see Figure 4d) and which contains several IS related elements (i.e. IS, transposases or pseudogenes of transposase). Events underlying rearrangements in this region cannot be exactly retraced but most likely they are ancient and have resulted in duplication of the *ptsG *gene in PG2 (MAG3250 and MAG3320). Interestingly, this region, like many others associated with IS, contains several genes or pseudogenes that have undergone HGT suggesting that IS may directly contribute to this phenomenon as suggested for other bacteria [43]. Finally, we showed that IS insertions may have an impact on gene expression, thus modifying some of the strain properties such as those associated with restriction-modification in 5632.

Compared to the PG2 type strain, 5632 seems better equipped for DNA exchange. Besides harbouring an impressive "mobilome", some of which may be tailored for conjugative transfer, it contains a number of operating RM systems. On one hand, these may act as a barrier to DNA invasion [44] and explain why 5632 DNA is resistant to several methylase-sensitive restriction enzymes and to DNA transformation (data not shown). On the other hand, while methylated DNA is protected against degradation, it might be more likely accepted by a recipient cell displaying similar RM systems, regardless of the DNA transfer or uptake mechanisms. Indeed, some of the 5632 specific RM systems not present in PG2 have homologs in members of the "mycoides" cluster (Table 2). Whether the structure of the *M. agalactiae *population is made of a majority of PG2-like strains that are deficient in mobile elements as well as in RM systems with only some strains such as 5632 being more prone to gene exchange with selected partners, is not yet known. Finally, DNA methylases, whether they belong or not to RM systems, could play a number of functions related to fitness or virulence, including the regulation of various physiological processes such as chromosome replication, mismatch repair, transposition, and transcription as described in other bacteria [45]. They may also be involved in the epigenetic switch of some key factors such as in the Pap of the uropathogenic *E. coli *[46].

Interestingly, a fairly good portion of the flexible gene pool of *M. agalactiae *is dedicated to producing surface proteins, many of which are lipoproteins. Based on *in silico *analysis, 5632 contains ca. 100 lipoproteins with at least 56 expressed under laboratory conditions. These include the Vpma family composed of 16 different lipoproteins that are encoded by 23 genes in 5632 and 6 in PG2 and that are phase variable in expression and probably in size [17]. Phase variation of surface molecules is a common mechanism in mycoplasma species [33] and is probably a major adaptive strategy for these minimal pathogens. Vpma phase variants are produced at high frequencies and in a reversible manner by site-specific recombination [26,47] but comparative proteogenomics conducted here suggest that other variable systems may co-exist. For instance, expression of the P48-like protein as a lipoprotein that is soluble in Triton-X114 may depend on a riboshifting mechanism or on reversible hypermutation in a polyG tract localised at the 5' coding sequence (Figure 6). Indeed, data obtained by Lynyansky *et al. *[48] showed that translation of a full length P68 lipoprotein in *M. bovis *is associated with the length of a similar polyG tract. The length of this homopolymer varies from 8 to 10 residues when comparing four *M. bovis *strains, with nine G allowing translation of a complete P68. Indeed, expression of the two homologs, the *M. agalactiae *P48-like and the *M. bovis *P68, is most likely phase variable in the two ruminant pathogens. Several other polyG tracts, some containing up to 13 residues, were found in the study that are associated with the 5' end of genes encoding surface lipoproteins suggesting that this may be a common slippage mechanism in *M. agalactiae*. Finally, the *drp *family involves genes that circulate by HGT between *M. agalactiae *and members of the "mycoides" cluster. Based on comparative proteogenomics, 5632 and PG2 have a same size repertoire each composed of a different set with only 2 out of 12 or 13 *drp *products being expressed, one common to the two strains and one specific. Whether this reflects a mechanism of phase variation is unlikely, but silent *drp *genes may act as a sequence reservoir for the emergence of new Drp expression patterns. Taken together these results suggest that the two *M. agalactiae *strains might display very different surface architectures with highly dynamic compositions during clonal propagation.

The strain 5632 was initially chosen because of its particular genetic features, several of which were found in its close relative *M. bovis*. This was further confirmed in this study which shows that 5632, unlike PG2, possesses (i) mobile elements such ICE and IS in multiple copies, (ii) a P48-like gene that is expressed, and (iii) two genes related to phage immunity that are also present in *M. bovis *PG45 [17]. The ovine/caprine pathogen *M. agalactiae *and the cattle pathogen *M. bovis *were first classified as the same species and our findings indicate that a continuum of strains might exist in between these two species. The genome sequence of *M. bovis *has been achieved (Craig Venter Institute, unpublished data and [31]) and its analysis may unravel even more common traits as well as some specificities that may explain their respective host-specificity.

## Conclusions

Multiple genome sequencing of closely related bacterial species can address various significant issues which range from a better understanding of forces driving microbial evolution to the design of novel vaccines.

Recent pan-genome studies using genome [3] or gene centred [49] approaches, strongly suggest that microbial genomes are continuously sampling and/or shuffling their genetic information rather than undergoing slow, progressive changes. By introducing the means for variability in the population, this dynamic process increases the chances for rapid adaptation and survival to changing environments.

Mycoplasma species have limited coding capacity yet our comparative study shows that two strains of the same species may display significant differences in the size of their mobile gene set, which one is marginal in the type strain but may represent up to 13% of a field strain. As observed for *E. coli*, these genes that relate to IS, phages or plasmids, are often associated with an accessory gene pool, usually represented by ORFans or genes present only in limited number of genomes across bacteria. For minimal genomes, this mobile gene set may provide a vehicle for the accessory as well as for the character genes to disseminate throughout population. Moreover, large mobile elements such as ICE may expand the genomic space, facilitating the emergence of new genes. This dynamic genome scheme may be crucial for mycoplasmas to counterbalance their reductive evolution so far marked by genome downsizing.

Ultra-high throughput genome sequencing is becoming more and more accessible so that wide and affordable studies will soon expand our knowledge of the mycoplasma pan-genome. Because several mycoplasma species are of importance for the medical and veterinary fields as well as excellent models for studying the minimal cell concept, this research area will undoubtly have a beneficial impact for both applied and theoretical mycoplasmology as well as for general microbiology.

## Methods

### Bacterial strains, culture conditions and DNA isolation

*M. agalactiae *type strain PG2, clone 55.5 [47] and strain 5632, clonal variant C1 [18] used in this study have been previously described. These strains have been independently isolated from goat in Spain. Experiments reported in this manuscript have all been performed with these clonal variants but for simplicity, we will further refer to them as PG2 and 5632. Mycoplasmas were propagated in complex Aluotto [50] or SP4 liquid medium [51] at 37°C and genomic DNA was extracted as described elsewhere [52,53].

### *M. agalactiae *strain 5632 sequencing and annotation

Whole genome sequencing of strain 5632 was performed as follows. A library of 3 kb inserts (A) was generated by mechanical shearing of the DNA followed by cloning of fragments into the pcDNA2.1 (Invitrogen) *E. coli *vector. Two libraries of 25 kb (B) and 80 kb (C) inserts also were generated by *Hin *dIII partial digestion and cloning of the resulting DNA fragments into the pBeloBAC11 (CALTECH) modified *E. coli *vector. The plasmid inserts of 10752, 3072 and 768 clones picked from the A, B and C libraries respectively were end-sequenced by dye-terminator chemistry on ABI3730 sequencer. The PHRED/PHRAP/CONSED software package was used for sequence assemblies. Gap closure and quality assessment were made according to the Bermuda rules with 10307 additional sequences. Annotation was performed as previously described using the CAAT-Box platform [54] with an automatic pre-annotation for CDS having a high similarity to PG2 followed by expert validation. Criteria used for the automatic pre-annotation step were: CDS considered as Probable IF %similarity >= 60 OR (IF %similary >= 35 AND START at identical position); Putative IF NOT Probable AND IF %similarity >= 35. Annotation fields were transposed IF (status = Probable OR Putative). The BLAST program suite was used for homology searches in non-redundant databases http://www.ncbi.nlm.nih.gov/blast/blast.cgi. In order to determine the extent of sequence similarity, alignments between sequences were performed using the Needle (Needleman-Wunsch global alignment algorithm) or the Water (Smith-Waterman local alignment algorithm) http://www.ebi.ac.uk/Tools/emboss/align/ software.

Lipoproteins were detected as previously described [12] based on the presence (i) of the PROSITE Prokaryotic membrane lipoprotein lipid attachment site motif (PROKAR_LIPOPROTEIN, Acc. Numb. PS00013) [DERK](6)-[LIVMFWSTAG](2)-[LIVMFYSTAGCQ]-[AGS]-C and/or (ii) of two motifs previously defined by MEME-MAST that correspond to a charged N-terminal followed by a specific lipobox. After manual confirmation, a total of 105 CDSs were annotated as predicted lipoproteins.

The tRNA genes were located on the chromosome using the tRNAscan-SE software [55]; the rRNA genes and the *rnpB *gene from the RNAseP system were searched using BLASTN by homology with *M. agalactiae *strain PG2 [12]; the tmRNA involved in translational surveillance and ribosome rescue was predicted using the ARAGORN software http://130.235.46.10/ARAGORN/[56].

Phylogenetic analyses were performed using MEGA 4.0 [57] and the Neighbor-Joining tree method. The reliability of the tree nodes was tested by performing 500 bootstrap replicates.

### Comparative genome analysis

Comparative genomic analyses involving *Mollicutes *genomes were performed using a combination of tools available in the MolliGen public database [58] after incorporating the 5632 genome into a private section. Other bioinformatics softwares were used that include (i) Artemis [59], (ii) Artemis Comparison Tool (ACT) [60], (iii) mVISTA [61].

### Proteomic analyses

After propagation of the two *M. agalactiae *strains respectively grown in the complex Aluotto media at 37°C, the cells were collected by centrifugation, washed three times in PBS before being re-suspended in this buffer. One aliquot was used for defining the total protein content (PG2) while the remaining was subjected to protein partitioning using Triton-Tx114 as previously described [62]. Partitioning resulted in three fractions corresponding to hydrophobic (suspended in Triton-TX114), hydrophilic and insoluble proteins that were further subjected to 1D SDS-PAGE as such except for the hydrophobic fraction which was first precipitated overnight at -70°C after addition of 9 volumes of cold MeOH and centrifugated 10 min at 12,000 × *g *and resuspended in loading buffer. The gel was sliced into about 15 sections which were subjected to trypsin digestion. Peptides were further analyzed by nano liquid chromatography coupled to a MS/MS ion-trap mass spectrometer (LC-MS/MS).

Peptides were identified with SEQUEST through the Bioworks 3.3.1 interface (Thermo-Finnigan, Torrence, CA, USA) against a database consisting of both direct and reverse sense *Mycoplasma agalactiae *strain PG2 or 5632 entries. Using the following criteria (DeltaCN ≥ 0.1, Xcorr vs Charge State ≥ 1.5 (+1), 2.0 (+2), 2.5 (+3), Peptide Probability ≤ 0.001 and Number of Different Peptides ≥ 2) as validation filters, the False Positive rate is null. Additional file 1: Table S1 summarizes the CDS which at least two specific peptides were detected in at least one of the fractions.

### Database submission and web-accessible database

The genome sequence from *M. agalactiae *strain 5632, as well as related features were submitted to the EMBL/GenBank/DDBJ databases under accession number FP671138. All data were also loaded into the MolliGen database http://cbi.labri.fr/outils/molligen/[58].

## Authors' contributions

LXN, PSP, MSM and CC carried out the genetic analyses, participated in manual expert annotation and drafted the manuscript. ES carried out mycoplasma cultures, genomic DNA extraction and proteins enrichment. SC carried out LC-MS/MS whole proteomic sequencing, and CC analysed the proteomic data. VB, CS and SM produced the DNA libraries and carried out genome sequencing, finishing and assembly. DJ and AB adapted the bioinformatics server for pre-annotation and analyses. MSM, PSP, AB and CC conceived and participated in the design of the study which was coordinated by CC. All authors read and approved the final manuscript.

## Supplementary Material

Additional file 1 **Table S1: Products of 5632 for which more than one specific peptide were detected by LC MS/MS after 1D SDS-PAGE**. List of the CDSs which expression was confirmed by proteomic data in 5632 and/or in PG2. Click here for file
