# Supplementary material for: Comparative genomic and proteomic analyses of two Mycoplasma agalactiae strains: clues to the macro- and micro-events that are shaping mycoplasma diversity
Source: BMC Genomics. 2010 Feb 2;11:86. doi: 10.1186/1471-2164-11-86 (PMC2824730; doi:10.1186/1471-2164-11-86)
Supplement: Additional file 1 — Table S1: Products of 5632 for which more than one specific peptide were detected by LC MS/MS after 1D SDS-PAGE. List of the CDSs which expression was confirmed by proteomic data in 5632 and/or in PG2. [file 1471-2164-11-86-S1.PDF]

Table S1: Products of 5632 for which more than one specific peptide were detected by LC MS/MS after 1D SDS-PAGE

MAGa[a]	Product	Detected by MS/MS in 5632[b]	Homolog detected in PG2[b]
MAGa0010	Chromosomal replication initiator protein DnaA	+	+
MAGa0020	DNApolymerase III, beta chain	+	+
MAGa0050	Esterase/lipase	+	-
MAGa0060	Esterase/lipase	-	+
MAGa0070	NADH dependent flavin oxidoreductase	-	-
MAGa0080	Lipoate-proteinligase A	+	-
MAGa0090	Lipoate-proteinligase A	+	+
MAGa0100	Conserved hypothetical protein	+	+
MAGa0110	Glycine cleavag system H protein	+	+
MAGa0120	Conserved hypothetical protein	-	-
MAGa0130	Hypothetical protein	+	-
MAGa0140	Conserved hypothetical protein, predicted lipoprotein, P48	+	+
MAGa0150	Hypothetical protein	-	-
MAGa0160	ABC transporter, ATP-binding protein, P59	+	+
MAGa0170	Sugar ABC transporter permease	+	+
MAGa0180	Sugar ABC transporter permease protein	-	-
MAGa0190	Deoxyguanosine kinase	+	+
MAGa0200	Deoxyguanosine kinase	+	-
MAGa0210	Conserved hypothetical protein, predicted lipoprotein	-	-
MAGa0220	Hypothetical protein, predicted lipoprotein,DUF285 family	-	-
MAGa0230	Conserved hypothetical protein, DUF285 family	-	-
MAGa0240	Transposase	-	-
MAGa0250	Hypothetical protein, predicted lipoprotein,DUF285 family	-	-
MAGa0260	Conserved hypothetical protein YeiN	+	+
MAGa0270	Conserved hypothetical protein YeiC	+	-
MAGa0280	Hydrolase of the HAD superfamily	+	-
MAGa0290	Conserved hypothetical protein	+	+
MAGa0300	Hypothetical ABC transporter, ATP-binding protein	+	-
MAGa0310	Hypothetical protein	-	-
MAGa0320	Hypothetical protein	-	-
MAGa0330	Hypothetical protein	-	-
MAGa0340	Oligopeptide ABC transporter, ATP-binding protein (OppF)	+	+
MAGa0350	Oligopeptide ABC transporter, ATP-binding protein (OppD)	+	+
MAGa0360	Oligopeptide ABC transporter, permease protein (OppC)	+	-
MAGa0370	Oligopeptide ABC transporter, permease protein (OppB)	+	-
MAGa0380	Oligopeptide ABC transporter, substrate-binding protein (OppA), predicted lipoprotein	+	+
MAGa0390	Conserved hypothetical protein	+	+
MAGa0400	Cysteinyl-tRNA synthetase (Cysteine-tRNA ligase)	+	-
MAGa0410	tRNA/rRNA methyltransferase	-	-
MAGa0420	Hypothetical protein	-	-
MAGa0430	50S ribosomal protein L33	-	-
MAGa0440	Hypothetical protein	-	-
MAGa0450	Transcription antitermination protein (NusG)	+	+
MAGa0460	Conserved hypothetical protein	-	-
MAGa0470	Uridylate kinase	+	+
MAGa0480	Ribosome recycling factor (Ribosome releasing factor)	+	+
MAGa0510	Conserved hypothetical protein	-	-
MAGa0520	Conserved hypothetical protein	-	-
MAGa0530	Glycerol-3-phosphate dehydrogenase [NAD(P)+]	+	-
MAGa0540	Dimethyladenosinetransferase (S-adenosylmethionine-6-N',N'-adenosyl(rRNA)dimethyltransferase) (16SrRNAdimethylase) (High level kasugamycin resistance protein ksgA) (Kasugamycin dimethyltransferase)	+	-
MAGa0550	Conserved hypothetical protein, Putative deoxyribonuclease (YabD) Deoxyribonuclease (TatD)	-	+
MAGa0560	tRNA modification GTPase (TrmE)	+	+
MAGa0570	Hypothetical protein	-	-
MAGa0580	Glyceraldehyde 3-phosphate dehydrogenase (GAPDH)	+	+
MAGa0590	Seryl-tRNA synthetase	+	+
MAGa0600	epsG	+	-
MAGa0610	50S ribosomal protein L34	-	-
MAGa0620	Ribonuclease P protein component	-	-
MAGa0630	Conserved hypothetical protein	+	+
MAGa0640	Lipoate-protein ligase A	+	+
MAGa0650	Esterase/lipase	+	+
MAGa0660	Hypothetical protein	-	-

MAGa0670	Aspartate-ammonialigase(AsnA)	+	-
MAGa0680	DNA polymerase III alpha subunit	+	-
MAGa0710	ATPase, AAA family	+	-
MAGa0720	Phenylalanyl-tRNA Synthetase alpha chain (PheS)	+	-
MAGa0730	Uracil-DNA glycosylase (Ung)	-	-
MAGa0740	Phenylalanyl-tRNA synthetase beta chain (PheT)	+	+
MAGa0750	Serine hydroxymethyltransferase (Serine methylase) (SHMT)	+	+
MAGa0760	Proline iminopeptidase (Pip)	-	-
MAGa0770	Nitrogen fixation proteinNifS	+	-
MAGa0780	Nitrogen fixation protein NifU	-	-
MAGa0790	DNA-damage repair protein MucB	+	-
MAGa0800	Bidomainal protein	+	+
MAGa0810	Ribosomal large subunit pseudouridine synthase B	+	+
MAGa0820	Hypothetical protein, predicted lipoprotein	-	-
MAGa0830	Hypothetical protein	+	-
MAGa0840	Fructose-bisphosphate aldolase (Fba)	+	+
MAGa0850	Nuclease	-	-
MAGa0860	50S ribosomal protein L11	+	+
MAGa0870	50S ribosomal protein L1	+	+
MAGa0880	Hypothetical protein	-	-
MAGa0890	Hypothetical protein	+	-
MAGa0900	Hypothetical protein	-	-
MAGa0910	tRNA/rRNA methyltransferase	+	+
MAGa0920	rRNA methylase	-	+
MAGa0930	Hypothetical protein	-	-
MAGa0940	GTP-binding protein	+	-
MAGa0950	Hypothetical protein	-	-
MAGa0960	HPrkinase/phosphorylase	+	+
MAGa0970	Prolipoprotein diacylglyceryl transferase	+	+
MAGa0980	Thioredoxinreductase	+	+
MAGa0990	Hypothetical protein	+	-
MAGa1000	Pyruvate dehydrogenase E1 component, alpha subunit	+	+
MAGa1010	Pyruvate dehydrogenase E1 component, beta subunit	+	+
MAGa1020	Hypothetical protein	+	-
MAGa1030	Dihydrolipoamide acetyltransferase component of pyruvate deshydrogenase complex	+	+
MAGa1040	Dihydrolipoamide dehydrogenase (E3 component of pyruvate complex)	+	+
MAGa1050	50S ribosomal protein L28	+	+
MAGa1060	Conserved hypothetical protein	-	-
MAGa1070	Conserved hypothetical protein	+	+
MAGa1080	Transposase	-	-
MAGa1090	Conserved hypothetical protein, predicted lipoprotein	+	+
MAGa1100	Oligopeptide ABC transporter, permease protein (OppB)	+	+
MAGa1110	Oligopeptide ABC transporter system, permease protein (OppC)	+	+
MAGa1120	Oligopeptide ABC transporter, ATP-binding protein(OppD)	+	+
MAGa1130	Oligopeptide ABC transporter, ATP-binding protein (OppF)	+	+
MAGa1140	Hypothetical protein, predicted lipoprotein	+	+
MAGa1150	Hypothetical protein	-	-
MAGa1160	Conserved hypothetical protein	+	-
MAGa1170	Hypothetical protein, predicted lipoprotein	-	-
MAGa1180	Ribose-5-phosphate isomerase	+	+
MAGa1190	Hypothetical protein	-	-
MAGa1200	Conserved hypothetical protein	-	-
MAGa1210	Conserved hypothetical protein	-	-
MAGa1220	Cytidine deaminase (Cytidine aminohydrolase)	-	+
MAGa1230	GTP-binding protein era	-	-
MAGa1240	XAA-PRO aminopeptidase	+	+
MAGa1250	Prolyl-tRNA synthetase (Proline-tRNA ligase)	+	+
MAGa1260	Hypothetical protein	-	-
MAGa1270	Hypothetical protein	+	+
MAGa1280	Conserved hypothetical protein	+	+
MAGa1290	Phosphoketolase	+	+
MAGa1300	Oligo endopeptidase F	+	+
MAGa1310	Spermidine/putrescine ABC transporter ATP-binding protein PotA	+	+
MAGa1320	Spermidine/putrescine ABC transporter permease protein PotB	-	-
MAGa1330	Spermidine/putrescine ABC transporter permease protein PotC	-	+
MAGa1340	Conserved hypothetica protein	+	+
MAGa1350	50S ribosomal protein L32	-	-
MAGa1360	Conserved hypothetical protein	+	-
MAGa1370	Thiamine biosynthesis protein thiI	+	-
MAGa1380	Hypothetical protein	-	+

MAGa1390	Conserved hypothetical protein, DUF285 family	-	-
MAGa1400	Conserved hypothetical protein	+	-
MAGa1410	Valyl-tRNA synthetase (Valine-tRNA ligase) (VALRS)	+	-
MAGa1420	FolD bifunctionalprotein (Methylene tetrahydrofolate Dehydrogenase; Methenyl tetrahydrofolate cyclohydrolase)	+	+
MAGa1430	Phosphate acetyltransferase (Phosphotransacetylase)	+	+
MAGa1440	Acetate kinase (Acetokinase)	+	+
MAGa1450	Phosphopantetheine adenylyltransferase (Pantetheine-phosphate adenylyltransferase) (PPAT) (Dephospho-CoA pyrophosphorylase)	-	-
MAGa1460	GTP-binding protein engB	-	-
MAGa1470	Hypothetical protein	+	+
MAGa1480	Pyruvate kinase (PK)	+	+
MAGa1490	Conserved hypothetical protein, predicted lipoprotein	+	+
MAGa1500	Chaperone protein dnaK	+	+
MAGa1510	Glucose-inhibited division protein	-	-
MAGa1520	Pseudogene	-	-
MAGa1530	D-lactate dehydrogenase	+	+
MAGa1540	Esterase/lipase	-	-
MAGa1550	Hypothetical protein, predicted lipoprotein	+	+
MAGa1560	Conserved hypothetical protein	+	+
MAGa1570	Type III restriction-modification system: Methylase	+	-
MAGa1580	Type III restriction-modification system: Methylase	+	+
MAGa1590	IS30-like protein	-	-
MAGa1600	Triggerfactor	+	+
MAGa1610	Hypothetical protein, predicted lipoprotein	-	+
MAGa1620	Conserved hypothetical protein, P48-like	+	-
MAGa1630	Hypothetical protein, predicted lipoprotein	-	-
MAGa1640	ABC transporter, ATP binding protein	-	+
MAGa1650	Conserved hypothetical protein, predicted membrane protein	-	-
MAGa1660	DNA polymerase I: 5'-3' exonuclease domain	+	-
MAGa1670	DNApolymerase III, alpha subunit	+	-
MAGa1680	Conserved hypothetical protein, predicted lipoprotein	+	+
MAGa1690	Hypothetical protein	-	-
MAGa1700	Hypothetical protein	-	-
MAGa1710	Hypothetical protein	-	-
MAGa1720	Hypothetical protein	-	-
MAGa1730	Glucose inhibited division proteinA	+	+
MAGa1740	Conserved hypothetical protein	-	-
MAGa1750	Conserved hypothetical protein, predicted transmembrane product	-	-
MAGa1760	Hypothetical protein	+	+
MAGa1770	DNA methylase	-	-
MAGa1780	Formylmethionine deformylase	+	+
MAGa1790	Hypothetical protein	+	+
MAGa1800	Topoisomerase IV subunit B	-	-
MAGa1810	Topoisomerase IV subunit A	+	+
MAGa1820	Conserved hypothetical protein	+	+
MAGa1830	Ribonuclease HII (RNase HII)	-	-
MAGa1850	Hypothetical protein	-	-
MAGa1870	Methyltransferase GidB (Glucose inhibited division protein B)	-	-
MAGa1880	Ribose-phosphate pyrophospho kinase (Phosphoribosyl pyrophosphate synthetase)	+	+
MAGa1890	Hypothetical protein, predicted lipoprotein	-	-
MAGa1900	Pseudogene	-	+
MAGa1910	Hypothetical protein, predicted lipoprotein	-	-
MAGa1920	Ribonuclease	+	-
MAGa1930	TransketolaseI	+	+
MAGa1940	Inorganic pyrophosphatase (Pyrophosphate phospho-hydrolase)	+	+
MAGa1950	30S ribosomal protein S20	+	+
MAGa1960	Hypothetical protein	-	+
MAGa1970	Conserved hypothetical protein	+	+
MAGa1980	Hypothetical protein, predicted lipoprotein	+	+
MAGa1990	HIT-likeprotein (Cell cycle regulation)	+	+
MAGa2000	Hypothetical protein, predicted lipoprotein	+	+
MAGa2010	50S ribosomal protein L33	-	-
MAGa2020	Thiol peroxidase	+	-
MAGa2030	Conserved hypothetical protein	-	-
MAGa2040	Cell division protein FtsY	+	+
MAGa2050	Hypothetical protein	+	+
MAGa2060	Methionyl-tRNA synthetase (Methionine-tRNA ligase)	+	+
MAGa2070	DNAmethylase	+	-
MAGa2080	RibonucleaseR (VacB-like(Shigella flexneri) ribonucleaseII)	+	+

MAGa2090	Hypothetical protein	-	-
MAGa2100	Guanylate kinase	+	+
MAGa2110	Protein phosphatase	-	-
MAGa2120	Serine/threonine-protein kinase	+	+
MAGa2130	GTPase EngC	-	-
MAGa2140	Ribulose-phosphate3-epimerase(Pentose-5-phosphate3-epimerase)	+	+
MAGa2150	Pseudogene of Exodeoxyribonuclease V alphachain(RecD) (C-terminal part)	-	-
MAGa2160	Transposase	-	-
MAGa2170	Pseudogene of Exodeoxyribonuclease V alphachain(RecD) (C-terminal part)	-	-
MAGa2180	Pseudogene of Exodeoxyribonuclease V alphachain(RecD) (N-terminal part)	-	-
MAGa2190	Conserved hypothetical protein	+	-
MAGa2200	AAA family ATPase	+	-
MAGa2210	Conserved hypothetical protein	+	-
MAGa2300	CTP synthase (UTP-ammonialigase)	+	+
MAGa2310	Isoleucyl-tRNA synthetase (Isoleucine-tRNA ligase)	+	+
MAGa2320	Lipoprotein signal peptidase (SPASE II)	+	+
MAGa2330	Conserved hypothetical protein, predicted lipoprotein	+	+
MAGa2390	Holliday junction DNA helicase ruvA	-	-
MAGa2400	Holliday junction DNA helicase ruvB	-	-
MAGa2410	Protein-export membrane protein	+	+
MAGa2420	tRNA pseudouridine synthase B	-	+
MAGa2430	Conserved hypothetical protein	+	+
MAGa2440	Riboflavin biosynthesis protein	-	-
MAGa2450	30S ribosomal protein S15	-	+
MAGa2460	Esterase/lipase	+	+
MAGa2470	Glycerol ABC transporter, ATP-binding component	+	-
MAGa2480	Glycerol ABC transporter, permease component	-	-
MAGa2490	Glycerol ABC transporter, permease component	-	-
MAGa2500	Conserved hypothetical protein, predicted lipoprotein	+	-
MAGa2510	Hypothetical protein, predicted lipoprotein	+	+
MAGa2520	Conserved hypothetical protein	+	+
MAGa2530	50SribosomalproteinL9	+	+
MAGa2540	ReplicativeDNAhelicase	+	+
MAGa2550	Hemolysin-related protein	+	-
MAGa2570	Hypothetical protein, predicted lipoprotein	+	+
MAGa2580	P40, predicted lipoprotein	+	+
MAGa2590	Hypothetical protein, predicted lipoprotein	-	-
MAGa2600	Conserved hypothetical protein, predicted lipoprotein, DUF285 family	+	+
MAGa2610	Hypotheticalprotein	+	-
MAGa2620	Hypothetical protein, predicted lipoprotein	-	-
MAGa2630	NADH oxidase (NOXASE)	-	+
MAGa2640	Proton/glutamate symporter	+	+
MAGa2650	DNA recombination protein	+	+
MAGa2660	Hypothetical protein	-	-
MAGa2670	Hypothetical protein, predicted lipoprotein	+	+
MAGa2680	Hypothetical protein, predicted lipoprotein	-	-
MAGa2690	Hypothetical protein, Vpma-like, predicted lipoprotein	+	-
MAGa2700	Adenine-specific DNA methyltransferase	-	-
MAGa2710	Type II restriction endonuclease	-	-
MAGa2720	30S ribosomal protein S2	+	+
MAGa2730	Elongation factor Ts (EF-Ts)	+	+
MAGa2740	Hypothetical protein, predicted lipoprotein	+	+
MAGa2750	Conserved hypothetical protein	-	-
MAGa2760	NADH oxidase (NOXASE)	+	+
MAGa2770	Conserved hypothetical protein	+	-
MAGa2780	DNA-directed RNA polymerase sigma factor(Sigma-A)	+	+
MAGa2790	DNA primase	+	-
MAGa2800	Glycyl-tRNA synthetase (Glycine-tRNAligase)	+	+
MAGa2810	Hypothetical protein	+	+
MAGa2820	Alkylphosphonate ABC transporter, substrate-binding protein, predicted lipoprotein	+	+
MAGa2830	ABC transporter, ATP-binding protein	-	-
MAGa2840	Alkylphosphonate ABC transporter, permease protein	-	-
MAGa2850	Conserved hypothetical protein	+	+
MAGa2860	Preprotein translocase SecA subunit	+	+
MAGa2870	Alcohol dehydrogenase	+	+
MAGa2880	DNA topoisomerase I	+	+
MAGa2890	30S ribosomal protein S6	+	+
MAGa2900	Single-stranded DNA-binding protein	-	+
MAGa2910	30S ribosomal protein S18	+	+
MAGa2920	Conserved hypothetical protein	-	-

MAGa2930	Phosphopento mutase	+	+
MAGa2940	Hypothetical protein	+	+
MAGa2950	DNA ligase	+	+
MAGa2960	Putative transmembrane protein	-	-
MAGa2970	Conserved hypothetical protein, predicted lipoprotein	+	+
MAGa2980	CDS1	-	-
MAGa2990	CDSA	-	-
MAGa3000	CDS12	-	-
MAGa3010	CDS 11	-	-
MAGa3030	CDSB	-	-
MAGa3040	CDSC	-	-
MAGa3050	CDSD	-	-
MAGa3060	CDS5	-	-
MAGa3070	CDS7	-	-
MAGa3080	CDS13	-	-
MAGa3090	CDS15	-	-
MAGa3100	Pseudogene of CDS16 (N-terminal part)	-	-
MAGa3110	Pseudogene of CDS16 (C-terminal part)	-	-
MAGa3120	Hypothetical protein	-	-
MAGa3130	CDS17	+	-
MAGa3140	CDS19	-	-
MAGa3150	CDSE	-	-
MAGa3160	CDS14	+	-
MAGa3170	CDSF	-	-
MAGa3180	Hypotetical protein	+	-
MAGa3190	CDSG	-	-
MAGa3200	CDSH	-	-
MAGa3210	Pseudogene of CDSG	-	-
MAGa3220	CDS22	-	-
MAGa3230	Conserved hypothetical protein, predicted transmembrane protein	+	-
MAGa3240	Putative transmembrane protein	-	-
MAGa3250	Conserved hypothetical protein, predicted lipoprotein	+	-
MAGa3260	Conserved hypothetical protein, predicted transmembrane protein	+	-
MAGa3270	Conservedhypotheticalprotein	+	+
MAGa3280	Conserved hypothetical protein	+	-
MAGa3290	Conservedhypotheticalprotein	+	-
MAGa3300	Putative transmembrane protein	+	+
MAGa3310	ATP synthase alpha chain	+	+
MAGa3320	ATPsynthasebetachain	+	+
MAGa3330	Hypothetical protein, predicted lipoprotein	-	+
MAGa3340	Hypothetical protein	-	-
MAGa3350	Conserved hypothetical protein, predicted lipoprotein	-	-
MAGa3360	Conserved hypothetical protein	+	+
MAGa3370	Conserved hypothetical protein	+	-
MAGa3380	ATP-dependenthelicase	+	-
MAGa3390	Conserved hypothetical protein	-	-
MAGa3400	Glycosyltransferase	+	-
MAGa3410	Asparaginyl-tRNA synthetase	+	+
MAGa3420	Hypothetical protein	-	-
MAGa3430	Hypothetical protein	-	-
MAGa3440	Conserved hypothetical protein	+	+
MAGa3450	Mg2+ transport protein (MGTE)	+	+
MAGa3460	Hypothetical protein	+	-
MAGa3470	Elongation factor P (EF-P)	+	+
MAGa3480	Hypothetical protein	+	+
MAGa3500	ABC transporter ATP-binding protein	+	+
MAGa3510	ABC transporter ATP-binding protein	+	+
MAGa3520	Conserved hypothetical protein	+	-
MAGa3530	Peptide methionine sulfoxide reductase	+	+
MAGa3540	Hypothetical protein	-	-
MAGa3550	Foramidopyrimidine DNA glycosylase	-	-
MAGa3560	Glucose-6-phosphate isomerase	+	-
MAGa3570	Glucose-6-phosphate isomerase	-	-
MAGa3580	30S ribosomal protein S1	-	-
MAGa3590	Enolase	+	+
MAGa3600	Elongation factor Tu (EF-Tu)	+	+
MAGa3610	Conserved hypothetical protein	-	-
MAGa3620	Conserved hypothetical protein	+	-
MAGa3630	O-sialoglycoprotein endopeptidase (Glycoprotease)	+	-
MAGa3640	Conserved hypothetical protein, predicted lipoprotein	+	+
MAGa3650	PtsG	+	+

MAGa3660	Conserved hypothetical protein	-	-
MAGa3670	Pseudogene	-	-
MAGa3680	Hypothetical protein	-	-
MAGa3690	Conserved hypothetical protein, truncated in C-terminal	-	-
MAGa3700	Transposase	-	-
MAGa3710	Pseudogene	-	-
MAGa3720	Hypothetical protein, truncated in N-terminal	-	-
MAGa3730	Hypothetical protein, predicted lipoprotein	-	-
MAGa3740	Hypothetical protein	-	-
MAGa3750	Pseudogene of Transposase	-	-
MAGa3760	Conserved hypothetical protein, DUF285 family, truncated in terminal	-	-
MAGa3770	Conserved hypothetical protein, DUF285 family	-	-
MAGa3780	Conserved hypothetical protein	+	-
MAGa3790	Tryptophanyl-tRNA synthetase	-	-
MAGa3800	Threonyl-tRNA synthetase	+	+
MAGa3810	Hypothetical protein	-	-
MAGa3820	Hypothetical protein, predicted lipoprotein	+	-
MAGa3830	P30, predicted lipoprotein	-	+
MAGa3840	Hypotheticalprotein	+	+
MAGa3850	Conserved hypothetical protein	-	-
MAGa3860	ATP synthase A chain	-	-
MAGa3870	ATP synthase C chain	-	-
MAGa3880	ATP synthase B chain	-	+
MAGa3890	ATP synthase delta subunit	+	+
MAGa3900	ATP synthase alpha chain	+	+
MAGa3910	ATP synthase gamma chain	-	-
MAGa3920	ATP synthase beta chain	+	+
MAGa3930	ATP synthase epsilon chain	-	-
MAGa3940	Hypothetical protein	+	-
MAGa3950	Cytosine-specific methyltransferase	+	-
MAGa3960	Transposase	-	-
MAGa3970	Type II site-specific deoxyribonuclease, *sau96*I-like	-	-
MAGa3980	Hypothetical protein, predicted lipoprotein	-	+
MAGa3990	Hypothetical protein, predicted lipoprotein	+	+
MAGa4000	Conserved hypothetical protein	-	-
MAGa4010	Hypotheticalprotein	+	-
MAGa4020	Hypothetical protein	-	-
MAGa4030	Transposase	-	-
MAGa4040	Conserved hypothetical protein, truncated inC-terminal	-	-
MAGa4050	Hypothetical protein	-	-
MAGa4060	Hypothetical protein	-	-
MAGa4070	Hypothetical protein	-	-
MAGa4080	Conserved hypothetical protein	-	-
MAGa4090	Heat shock ATP-dependentprotease	+	+
MAGa4100	Leucyl-tRNA synthetase (Leucine-tRNA ligase)	+	+
MAGa4110	Conserved hypothetical protein	-	-
MAGa4120	Uracil phosphoribosyl transferase	+	+
MAGa4130	Single-strand binding protein (Helix-destabilizing protein)	+	+
MAGa4140	Endopeptidase O	+	+
MAGa4150	Conserved hypothetical protein	+	-
MAGa4160	Glycine cleavage system H protein	-	+
MAGa4170	Hypothetical protein	-	-
MAGa4180	Cell division protein ftsZ	+	-
MAGa4190	Conserved hypothetical protein	-	-
MAGa4200	S-adenosyl-methyltransferase	-	-
MAGa4210	Protein MraZ	-	-
MAGa4220	Potassium uptake protein KtrA	+	+
MAGa4230	Potassium uptake protein KtrB	-	-
MAGa4240	Hypothetical protein	-	+
MAGa4250	ModificationmethylaseBsp6I	+	-
MAGa4260	Type II restriction enzyme Bsp6I	+	-
MAGa4270	UvrABCsystem protein B	+	+
MAGa4280	UvrABC system protein A	+	+
MAGa4290	Conserved hypothetical protein	+	+
MAGa4300	Hypothetical protein	-	-
MAGa4310	GTP-binding protein	-	+
MAGa4330	Hypothetical protein	+	-
MAGa4340	Hypoxanthine-guanine phosphoribosyl transferase(HGPRT)	-	-
MAGa4350	Transcription elongation factor greA	+	+
MAGa4360	Oxidoreductase	+	-
MAGa4370	Conserved hypothetical protein	+	+

Table S1 continued 6/12

MAGa4380	Conserved hypothetical protein	+	+
MAGa4390	Putative Holliday junction resolvase	-	-
MAGa4400	Alanyl-tRNA synthetase (Alanine-tRNA ligase)(ALARS)	+	+
MAGa4410	tRNA (5-methylaminomethyl-2-thiouridylate)-methyltransferase	-	-
MAGa4420	Purine nucleoside phosphorylase (Inosine phosphorylase) (PNP)	+	+
MAGa4430	Hypothetical protein	-	-
MAGa4440	Conserved hypothetical protein	+	-
MAGa4450	Conserved hypothetical protein, DUF285 family	-	+
MAGa4460	Hypothetical protein, predicted lipoprotein	-	-
MAGa4470	Pseudogene of CpG DNA methylase (N-terminal part)	-	-
MAGa4480	Pseudogene of CpG DNA methylase (C-terminal part)	-	-
MAGa4490	Conserved hypothetical protein	-	-
MAGa4500	Hypothetical protein, predicated lipoprotein	-	-
MAGa4510	Alcohol dehydrogenase	-	+
MAGa4520	Hypothetical protein, predicted lipoprotein	-	-
MAGa4530	Hypothetical protein, DUF285 family	-	-
MAGa4540	Conserved hypothetical protein, predicted lipoprotein, DUF285 family	-	-
MAGa4550	Hypothetical protein, predicted lipoprotein	-	-
MAGa4560	Alcohol dehydrogenase	+	+
MAGa4570	DNA processing protein (Smf)	-	-
MAGa4580	Hydrolase	+	+
MAGa4590	ABC transporter permease protein	+	-
MAGa4600	P115-Like (Mycoplasma hyorhinis) ABC transporter ATP-Binding Protein	+	+
MAGa4610	Ribonuclease III (RNase III)	-	-
MAGa4620	Fatty acid/phospholipid synthesis protein (PlsX)	+	+
MAGa4630	Conserved hypothetical protein	+	+
MAGa4640	Hypothetical protein	-	+
MAGa4650	Conserved hypothetical protein	-	-
MAGa4660	Conserved hypothetical protein (MgpA-like)	+	+
MAGa4670	Conserved hypothetical protein (MgpA-like)	+	+
MAGa4680	Conserved hypothetical protein, predicted lipoprotein	+	+
MAGa4690	Glycerol kinase	+	+
MAGa4700	Glycerol facilitator factor	+	+
MAGa4710	Aspartyl-tRNA synthetase (Aspartate-tRNA ligase) (AspRS)	+	+
MAGa4720	Histidyl-tRNA synthetase (Histidine-tRNA ligase) (HisRS)	+	+
MAGa4730	Conserved hypothetical protein	+	-
MAGa4740	Conserved hypothetical protein	-	-
MAGa4750	Hypothetical protein	+	+
MAGa4760	Conserved hypothetical protein	+	-
MAGa4770	50S ribosomal protein L13	+	+
MAGa4780	30S ribosomal protein S9	+	+
MAGa4800	Conserved hypothetical protein	-	-
MAGa4810	UTP-glucose-1-phosphateuridyl transferase	+	+
MAGa4820	Transposase	-	-
MAGa4840	Cation-transporting P-type ATPase	+	+
MAGa4850	CDS1	-	-
MAGa4860	CDSA	-	-
MAGa4870	CDS12	-	-
MAGa4880	CDS11	-	-
MAGa4890	CDSB	-	-
MAGa4900	CDSC	-	-
MAGa4910	CDSD	-	-
MAGa4920	CDS5	-	-
MAGa4930	CDS7	-	-
MAGa4940	CDS13	-	-
MAGa4950	CDS15	-	-
MAGa4960	CDS16	-	-
MAGa4970	Hypothetical product	-	-
MAGa4980	CDS17	+	-
MAGa4990	CDS19	-	-
MAGa5000	CDSE	-	-
MAGa5010	CDS14	+	-
MAGa5020	CDSF	-	-
MAGa5030	Hypotetical protein	+	-
MAGa5040	CDSG	-	-
MAGa5050	CDSH	-	-
MAGa5060	CDS22	-	-
MAGa5070	ABC transporter, permease protein	+	+
MAGa5080	ABC transporter, ATP-binding protein	+	+
MAGa5090	Conserved hypothetical protein	-	-
MAGa5100	Conserved hypothetical protein	-	+

MAGa5110	Conserved hypothetical protein, predicted lipoprotein	-	+
MAGa5120	Phosphomannomutase	+	+
MAGa5130	Conserved hypothetical protein	-	-
MAGa5140	Transcription termination factor (NusB)	+	+
MAGa5150	Acylcarrier protein phosphodiesterase	+	+
MAGa5160	Phosphocarrier protein HPr (Histidine-containing protein)	+	+
MAGa5170	Oligopeptide transport system permease protein (OppB)	-	-
MAGa5180	Conserved hypothetical protein	-	-
MAGa5190	Conserved hypothetical protein, predictedlipoprotein	+	-
MAGa5200	Conserved hypothetical protein	-	-
MAGa5210	Hypothetical protein, predicted lipoprotein	+	+
MAGa5220	Translation initiation factor IF-3	+	+
MAGa5230	50S ribosomal protein L35	-	-
MAGa5240	50S ribosomal protein L20	+	+
MAGa5250	ATP-binding protein	+	-
MAGa5260	Hypothetical protein	-	+
MAGa5270	LicA	+	-
MAGa5280	GTP-binding protein (LepA)	+	-
MAGa5290	Conserved hypothetical protein	+	-
MAGa5300	NADPH flavin oxidoreductase	+	+
MAGa5310	Esterase/lipase	+	-
MAGa5320	Pseudogene of Polysaccharide synthase (N-terminal part)	-	-
MAGa5330	Transposase	-	-
MAGa5340	Pseudogene of Polysaccharide synthase (C-terminal part)	-	-
MAGa5350	Arginyl-tRNA synthetase	+	+
MAGa5360	Malate permease	+	+
MAGa5370	L-lactate dehydrogenase (L-LDH)	+	+
MAGa5380	Hypothetical protein	+	+
MAGa5390	Phosphate acetyl transferase (Phosphotransacetylase)	+	+
MAGa5400	Acetatekinase(Acetokinase)	+	+
MAGa5410	Transmembrane protein	-	+
MAGa5420	Conserved hypothetical protein, predicted lipoprotein	+	-
MAGa5430	Hexose phosphate transport protein	+	+
MAGa5440	Conserved hypothetical protein	+	-
MAGa5450	EndonucleaseIV	+	+
MAGa5460	Hypothetical protein	-	-
MAGa5470	Thioredoxin (TRX)	-	-
MAGa5480	UvrABC system protein C	-	-
MAGa5490	Hypothetical protein, predicted lipoprotein	+	-
MAGa5500	P80, predicted lipoprotein	+	+
MAGa5510	Conserved hypothetical protein, predictedl ipoprotein	+	+
MAGa5520	ABC transporter ATP-binding protein	+	+
MAGa5530	ABC transporter permease protein	+	+
MAGa5540	ABC transporter permease protein	+	+
MAGa5550	Hypothetical protein	-	-
MAGa5560	Hypothetical protein, predicted lipoprotein	+	+
MAGa5570	GTP-binding protein	+	+
MAGa5580	Conserved hypothetical protein	+	+
MAGa5590	Purine nucleoside phosphorylase (Inosine phosphorylase)	+	+
MAGa5600	Thymidine phosphorylase (TDRPASE)	+	+
MAGa5610	Deoxyribose-phosphatealdolase (Phosphodeoxyriboaldolase) (Deoxyriboaldolase)	+	+
MAGa5620	Triose phosphate isomerase	+	+
MAGa5630	Hypothetical protein, predicted lipoprotein	+	+
MAGa5640	IS30-like protein	-	-
MAGa5650	ABC transporter permease protein	+	+
MAGa5660	ABC transporter ATP-binding protein	+	+
MAGa5670	ABC transporter ATP-binding protein	+	+
MAGa5680	50S ribosomal protein L17	+	+
MAGa5690	DNA-directed RNA polymerase alpha chain (RNAP alpha subunit) (Transcriptase alpha chain) (RNA polymerase alpha subunit)	+	+
MAGa5700	30S ribosomal protein S11	+	+
MAGa5710	30S ribosomal protein S13	+	+
MAGa5720	Translation initiation factor IF-1	+	+
MAGa5730	Methionine aminopeptidase (MAP)	+	+
MAGa5740	Adenylatekinase (ATP-AMP transphosphorylase)	+	+
MAGa5750	Preprotein translocase SecY subunit	+	+
MAGa5800	Transposase	-	-
MAGa5810	Variable surface lipoprotein A (VpmaA)	+	-
MAGa5820	Variable surface lipoprotein B (VpmaB)	+	-
MAGa5830	Variable surface lipoprotein C (VpmaC)	+	-

MAGa5840	Variable surface lipoprotein D1 (VpmaD1)	+	-
MAGa5850	Variable surface lipoprotein E (VpmaE)	+	-
MAGa5860	Variable surface lipoprotein F1 (VpmaF1)	+	-
MAGa5870	Variable surface lipoprotein D2 (VpmaD2)	+	-
MAGa5880	Integrase-recombinase	-	-
MAGa5890	Transposase	-	-
MAGa5900	Hypothetical protein	-	-
MAGa5910	Hypothetical protein	-	-
MAGa5920	50S ribosomal protein L15	+	+
MAGa5930	30S ribosomal protein S5	+	+
MAGa5940	50S ribosomal protein L18	+	+
MAGa5950	50S ribosomal protein L6	+	+
MAGa5960	30S ribosomal protein S8	+	+
MAGa5970	30S ribosomal protein S14	-	-
MAGa5980	50S ribosomal protein L5	+	+
MAGa5990	50S ribosomal protein L24	+	+
MAGa6000	50S ribosomal protein L14	+	+
MAGa6010	30S ribosomal protein S17	+	+
MAGa6020	50S ribosomal proteinL29	+	+
MAGa6030	50S ribosomal proteinL16	+	+
MAGa6040	30S ribosomal protein S3	+	+
MAGa6050	50S ribosomal protein L22	+	+
MAGa6060	30S ribosomal protein S19	+	+
MAGa6070	50S ribosomal protein L2	+	+
MAGa6080	50S ribosomal protein L23	+	+
MAGa6090	50S ribosomal protein L4	+	+
MAGa6100	50S ribosomal protein L3	+	+
MAGa6110	30S ribosomal protein S10	+	+
MAGa6120	DNA-binding histone-like protein HU	+	+
MAGa6130	Putative recombinase protein	-	-
MAGa6140	RecA protein	+	-
MAGa6150	Conserved hypothetical protein	-	+
MAGa6160	Aminopeptidase	+	+
MAGa6170	16S rRNA uridine-516 pseudouridylate synthase and related pseudouridylate synthase	-	-
MAGa6180	50S ribosomal protein L27	-	+
MAGa6190	50S ribosomal protein L21	+	+
MAGa6200	Conserved hypothetical protein	-	-
MAGa6210	Large-conductance mechanosensitive channel	+	+
MAGa6220	Heat shock protein GrpE (activation of DnaK)	+	+
MAGa6230	Heat-inducible transcription repressor hrcA	+	-
MAGa6240	50S ribosomal protein L31	-	-
MAGa6250	30S ribosomal protein S4	+	+
MAGa6260	Hypothetical protein	-	-
MAGa6270	DNAgyrasesubunitA	+	+
MAGa6280	Type I R/M system specificity subunit	-	-
MAGa6290	Modification (Methylase) protein of type I restriction-modification system	+	-
MAGa6300	Conserved hypothetical protein	-	-
MAGa6310	Type I R/M system specificity subunit	-	+
MAGa6320	Phage family integrase	-	-
MAGa6330	HsdR, Restriction-modification enzyme subunitR	+	-
MAGa6340	Type I R/M system specificity subunit	+	-
MAGa6350	Modification (Methylase) protein of type I restriction-modification system HsdM	+	+
MAGa6360	Hypothetical protein, predicted lipoprotein	-	-
MAGa6370	Pseudogene	-	-
MAGa6380	5-formyltetrahydrofolate cyclo-ligase	-	-
MAGa6390	Conserved hypothetical protein	-	-
MAGa6400	Hypothetical protein, predicted lipoprotein	-	-
MAGa6410	Glutamyl-tRNAsynthetase	+	+
MAGa6420	Conserved hypothetical protein	-	-
MAGa6430	Methionine adenosyltransferase	+	-
MAGa6440	Conserved hypothetical protein	-	-
MAGa6450	Signal Recognition Particle Protein	+	+
MAGa6460	Acetyltransferase	+	+
MAGa6470	Hypotheticalprotein	+	-
MAGa6480	Conserved hypothetical protein	+	+
MAGa6490	Phosphoglycerate kinase	+	+
MAGa6510	Hypothetical protein	-	-
MAGa6520	Hypothetical protein	-	-
MAGa6530	Hypothetical protein	-	-

MAGa6540	Aminopeptidase	+	-
MAGa6550	Hypothetical protein, predicted lipoprotein	-	-
MAGa6560	5' Nucleotidase, predicted lipoprotein	+	+
MAGa6570	Elongation factor G	+	+
MAGa6580	30S ribosomal protein S7	+	+
MAGa6590	30S ribosomal protein S12	+	+
MAGa6600	Conserved hypothetical protein	+	+
MAGa6610	ABC transporter ATP-Binding Protein	+	+
MAGa6620	Hypothetical protein	-	-
MAGa6650	ABC transporter, ATP-binding protein	+	+
MAGa6660	ABC transporter, ATP-binding protein	+	+
MAGa6670	Hypothetical protein, predicted lipoprotein	-	-
MAGa6680	Hypothetical protein	-	-
MAGa6690	Hypothetical protein	-	-
MAGa6700	Hypothetical protein	-	-
MAGa6710	Hypothetical protein	-	-
MAGa6720	Hypothetical product, predicted lipoprotein	-	-
MAGa6730	Hypothetical protein, predicted lipoprotein	-	-
MAGa6740	Hypothetical protein	-	-
MAGa6750	Hypothetical protein, predicted lipoprotein	-	-
MAGa6760	Hypothetical protein	-	-
MAGa6770	Hypothetical protein, predicted lipoprotein	-	-
MAGa6780	Hypothetical protein, predicted lipoprotein	-	-
MAGa6790	Hypothetical protein	-	-
MAGa6800	Hypothetical protein	-	-
MAGa6810	Aminopeptidase	+	+
MAGa6820	Hypothetical protein, predicted lipoprotein	-	-
MAGa6830	Conservedhypotheticalprotein	+	-
MAGa6840	DNA-directed RNA polymerase beta' chain	+	+
MAGa6850	DNA-directed RNA polymerase beta chain	+	+
MAGa6860	Hypothetical protein, predicted lipoprotein	-	-
MAGa6870	Hypothetical protein	-	-
MAGa6880	CDS22	-	-
MAGa6890	Pseudogene of CDSG	-	-
MAGa6900	CDSH	-	-
MAGa6910	CDSG	-	-
MAGa6920	Hypothetical protein	+	-
MAGa6930	CDSF	-	-
MAGa6940	CDS14	+	-
MAGa6950	CDSE	-	-
MAGa6960	CDS19	-	-
MAGa6970	CDS17	+	-
MAGa6980	Hypothetical protein	-	-
MAGa6990	CDS16	-	-
MAGa7000	CDS15	-	-
MAGa7010	CDS13	-	-
MAGa7020	CDS7	-	-
MAGa7030	CDS5	-	-
MAGa7040	CDSD	-	-
MAGa7050	CDSC	-	-
MAGa7060	CDSB	-	-
MAGa7070	CDS11	-	-
MAGa7080	CDS12	-	-
MAGa7090	CDSA	-	-
MAGa7100	CDS1	-	-
MAGa7110	Pseudogene of Transposase	-	-
MAGa7120	Pseudogene of Transposase	-	-
MAGa7130	Hypothetical protein, predicted lipoprotein	+	+
MAGa7140	50SribosomalproteinL7/L12	+	+
MAGa7150	50S ribosomal protein L10	+	+
MAGa7160	Hypothetical protein, predicted lipoprotein	+	+
MAGa7170	Lysyl-tRNA synthetase	+	+
MAGa7180	Conserved hypothetical protein	+	+
MAGa7190	Conserved hypothetical protein	+	+
MAGa7200	ClpB	+	+
MAGa7210	30S ribosomal protein S16	+	+
MAGa7220	tRNA (Guanine-N(1)-)-methyltransferase	-	+
MAGa7230	50S ribosomal protein L19	+	+
MAGa7240	Methionyl-tRNA formyl transferase	+	+
MAGa7250	Conserved hypothetical protein	+	-
MAGa7260	Phosphoenolpyruvate-protein phosphotransferase	-	+

MAGa7270	Transcriptional regulator	+	-
MAGa7280	Conserved hypothetical protein	-	-
MAGa7290	SugarisomeraseSGAE	+	+
MAGa7300	Hexulose-6-phosphate isomerase (HUMPI)	-	+
MAGa7310	Hexulose-6-phosphate synthase (HUMPS)	-	+
MAGa7320	Pentitol phosphotransferase enzyme II, A component	-	-
MAGa7330	Pentitol phosphotransferase enzyme II, B component	-	+
MAGa7340	Transport protein SGAT	+	+
MAGa7350	Conserved hypothetical protein	+	+
MAGa7360	Conserved hypothetical protein	+	+
MAGa7370	Conserved hypothetical protein	-	-
MAGa7380	Hypothetical protein	-	-
MAGa7390	Hypothetical protein	-	-
MAGa7400	CDSE	-	-
MAGa7410	Conserved hypothetical protein	-	-
MAGa7420	IS30-like protein	-	-
MAGa7430	Hypothetical protein, predicted lipoprotein	-	-
MAGa7440	Hypothetical protein, predicted lipoprotein	-	-
MAGa7450	Conserved hypothetical protein, predictedl ipoprotein, DUF285 family, truncated in N-terminal	-	-
MAGa7460	Conserved hypothetical protein, Fic protein family	-	-
MAGa7470	Hypothetical protein, predicted lipoprotein,DUF285 family	+	-
MAGa7480	Hypothetical protein	-	-
MAGa7490	Conserved hypothetical protein, predicted lipoprotein	+	+
MAGa7500	Glycerol ABC transporter, permease component	-	-
MAGa7510	Glycerol transporter subunit B	-	-
MAGa7520	Glycerol ABC transporter, ATP-binding component	+	-
MAGa7530	Conserved hypothetical protein	-	+
MAGa7540	Conserved hypothetical protein	-	-
MAGa7550	Conserved hypothetical protein	+	+
MAGa7560	NH(3)-dependent NAD(+) synthetase	-	+
MAGa7570	ICEF-IA ORF9 ortholog	+	-
MAGa7580	Esterase/lipase	+	-
MAGa7590	Hypothetical RNA methyltransferase	-	-
MAGa7600	PutativeDNAhelicase	+	-
MAGa7610	Conserved hypothetical protein	-	-
MAGa7620	Conserved hypothetical protein	-	-
MAGa7630	Conserved hypothetical protein	-	-
MAGa7650	Modification methylase	+	-
MAGa7660	Hypothetical protein	+	-
MAGa7670	Conserved hypothetical protein	+	-
MAGa7680	Hypothetical protein	-	-
MAGa7690	Ribonuclease H II	-	-
MAGa7700	Conserved hypothetical protein	+	+
MAGa7710	Ribosomal large subunit pseudouridine synthase D	+	+
MAGa7720	Chromate transport protein	-	-
MAGa7730	Chromate transport protein	-	+
MAGa7740	tRNA(guanine-N(7)-)-methyltransferase	+	+
MAGa7750	Conserved hypothetical protein	-	+
MAGa7760	Conserved hypothetical protein	-	-
MAGa7770	Conserved hypothetical protein	+	-
MAGa7780	Hypothetical protein	-	-
MAGa7790	Conserved hypothetical protein	-	-
MAGa7800	DNA polymerase III subunit	-	-
MAGa7810	Thymidylate kinase	+	+
MAGa7820	Recombination protein RecR	-	+
MAGa7830	Hypothetical protein	-	-
MAGa7840	DNApolymerase III subunit gamma and tau	+	+
MAGa7850	Hypothetical protein	+	-
MAGa7860	2,3-bisphosphoglycerate-independent phosphoglycerate mutase	+	+
MAGa7870	Conserved hypothetical protein	-	-
MAGa7880	Hypothetical protein	+	-
MAGa7900	Conserved hypothetical protein	+	+
MAGa7910	CDP-diacylglycerol-glycerol-3-phosphate 3-phosphatidyl transferase	-	-
MAGa7920	Phosphatidate cytidylyl transferase	-	-
MAGa7930	Transposase	-	-
MAGa7940	Conserved hypothetical protein	-	-
MAGa7950	Adenine phosphoribosyltransferase	-	+
MAGa7960	Translation initiation factor IF-2	+	+
MAGa7970	Conserved hypothetical protein	-	-
MAGa7980	Transcription elongation protein NusA	+	+

MAGa7990	Conservedhypotheticalprotein	+	-
MAGa8000	Thymidine kinase	+	+
MAGa8010	Aminopeptidase (Leucine aminopeptidase)	+	+
MAGa8020	Conserved hypothetical protein	+	-
MAGa8030	Hypothetical protein	-	-
MAGa8040	Variable surface lipoprotein G (VpmaG)	+	-
MAGa8050	Variable surface lipoprotein F2 (VpmaF2)	+	-
MAGa8060	Variable surface lipoprotein X (VpmaX)	+	+
MAGa8070	Variable surface lipoprotein W (VpmaW)	+	+
MAGa8080	Variable surface lipoprotein C (VpmaC)	+	-
MAGa8090	Variable surface lipoprotein E (VpmaE)	+	-
MAGa8100	Variable surface lipoprotein B (VpmaB)	+	-
MAGa8110	Variable surface lipoprotein A (VpmaA)	+	-
MAGa8120	Variable surface lipoprotein D2 (VpmaD2)	+	-
MAGa8130	Conserved hypothetical protein	+	-
MAGa8140	Conserved hypothetical protein	-	-
MAGa8150	Variable surface lipoprotein H (VpmaH)	+	-
MAGa8160	Variable surface lipoprotein I (VpmaI)	+	-
MAGa8170	Variable surface lipoprotein F1 (VpmaF1)	+	-
MAGa8180	Variable surface lipoprotein J (VpmaJ)	+	-
MAGa8190	Variable surface lipoprotein K (VpmaK)	-	-
MAGa8200	Variable surface lipoprotein L (VpmaL)	-	-
MAGa8210	Variable surface lipoprotein D1 (VpmaD1)	+	-
MAGa8220	Integrase-recombinase	-	-
MAGa8230	Transposase	-	-
MAGa8250	Conserved hypothetical protein	+	+
MAGa8260	Hypothetical protein, predicted lipoprotein	+	-
MAGa8270	Conserved hypothetical protein	-	-
MAGa8280	Hypothetical protein, predicted lipoprotein	-	-
MAGa8290	Aspartyl/glutamyl-tRNA (Asn/Gln) amidotransferase subunit	+	+
MAGa8300	Glutamyl-tRNA (Gln) amidotransferase subunit A	-	+
MAGa8310	Conserved hypothetical protein	-	-
MAGa8320	Ribosomal large subunit pseudouridine synthase C	+	-
MAGa8330	Segregation and condensation protein B	+	+
MAGa8340	Segregation and condensation protein A	+	-
MAGa8350	1-acyl-SN-glycerol-3-phosphate acyltransferase	+	+
MAGa8360	Holo-[acyl-carrier protein]synthase	-	-
MAGa8370	GTP-binding protein engA	+	+
MAGa8380	Cytidylatekinase(CK)	+	+
MAGa8390	Hypothetical protein	-	-
MAGa8400	Conserved hypothetical protein, predicted lipoprotein, DUF285 family	-	-
MAGa8410	Pseudogene	-	-
MAGa8420	Heat shock protein DNAJ (activation of DnaK)	+	+
MAGa8440	Ribosome-binding factor A	-	-
MAGa8460	Tyrosyl-tRNA synthetase 1	+	+
MAGa8470	Hypothetical protein	-	+
MAGa8480	Conserved hypothetical protein	+	+
MAGa8500	Acyl carrier protein homolog	-	-
MAGa8510	Protoporphirogen oxidase HEMK	-	-
MAGa8520	Peptide chain release factor 1	+	+
MAGa8530	Conserved hypothetical protein	+	+
MAGa8540	DNA gyrase subunit B	+	+
MAGa8550	Thioredoxin	+	+
MAGa8560	SsrA-binding protein	-	-
MAGa8570	Conserved hypothetical protein	+	+
MAGa8580	Hypothetical protein	-	-
MAGa8600	Cation-transporting P-ATPase	+	+
MAGa8610	ABC transporter ATP-binding protein	+	+
MAGa8620	ABC transporter permease protein	+	+
MAGa8630	Cell division protein ftsH homolog	+	+
MAGa8640	Conserved hypothetical protein	-	-
MAGa8650	Peptidyl-tRNA hydrolase	+	+
MAGa8660	Exodeoxyribonuclease V alpha chain	+	-

[a], CDS of *M. agalactiae* strain 5632 (Molligen Mnemonic)

[b], Proteomic (see the Methods section) : (+) indicates that at least two peptides were detected by MS/MS for the corresponding CDS, suggesting the expression of the corresponding gene, (-) indicates that 0 to 1 specific peptide were detected for the corresponding CDS and that it was not confirmed as being expressed under our conditions.
